# Inducible LGALS3BP/90K activates antiviral innate immune responses by targeting TRAF6 and TRAF3 complex

**DOI:** 10.1371/journal.ppat.1008002

**Published:** 2019-08-12

**Authors:** Gang Xu, Zhangchuan Xia, Feiyan Deng, Lin Liu, Qiming Wang, Yi Yu, Fubing Wang, Chengliang Zhu, Weiyong Liu, Zhikui Cheng, Ying Zhu, Li Zhou, Yi Zhang, Mengji Lu, Shi Liu

**Affiliations:** 1 State Key Laboratory of Virology, Modern Virology Research Center, College of Life Sciences, Wuhan University, Wuhan, China; 2 College of Bioscience and Biotechnology, Hunan Agricultural University, Changsha, Hunan Province, China; 3 The Key Laboratory of Biosystems Homeostasis and Protection of the Ministry of Education and Innovation Center for Cell Signaling Network, Life Sciences Institute, Zhejiang University, Hangzhou, Zhejiang, China; 4 Department of Laboratory Medicine, Zhongnan Hospital of Wuhan University, Wuhan, China; 5 Department of Clinical Laboratory, Renmin Hospital of Wuhan University, Wuhan, Hubei, China; 6 Department of Clinical Laboratory, Tongji Hospital, Tongji Medical College, Huazhong University of Science and Technology, Wuhan, China; 7 Animal Biosafety Level III Laboratory at the Center for Animal Experiment, School of Medicine, Wuhan University, Wuhan, China; 8 Hubei Provincial Cooperative Innovation Center of Industrial Fermentation, College of Food and Pharmaceutical Engineering, Hubei University of Technology, Wuhan, China; 9 Institute of Virology, University Hospital of Essen, University of Duisburg-Essen, Essen, Germany; Emory University School of Medicine, UNITED STATES

## Abstract

The galectin 3 binding protein (LGALS3BP, also known as 90K) is a ubiquitous multifunctional secreted glycoprotein originally identified in cancer progression. It remains unclear how 90K functions in innate immunity during viral infections. In this study, we found that viral infections resulted in elevated levels of 90K. Further studies demonstrated that 90K expression suppressed virus replication by inducing IFN and pro-inflammatory cytokine production. Upon investigating the mechanisms behind this event, we found that 90K functions as a scaffold/adaptor protein to interact with TRAF6, TRAF3, TAK1 and TBK1. Furthermore, 90K enhanced TRAF6 and TRAF3 ubiquitination and served as a specific ubiquitination substrate of TRAF6, leading to transcription factor NF-κB, IRF3 and IRF7 translocation from the cytoplasm to the nucleus. Conclusions: 90K is a virus-induced protein capable of binding with the TRAF6 and TRAF3 complex, leading to IFN and pro-inflammatory production.

## Author summary

The innate immune system detects the presence of viruses through germline-encoded pattern-recognition receptors (PRRs) and leads to the production of proinflammatory cytokines and interferons (IFNs) as the first line of defense against viral infections. Here, we identified a host protein, LGALS3BP, as a positive regulator of PRR-mediated signal transduction pathways by interacting with TRAF6-TAK1 and TRAF3-TBK1 axes, enhancing their recruitment and promoting the ubiquitination of TRAF6 and TRAF3. LGALS3BP exhibited antiviral activity toward a broad range of viral infections. LGALS3BP^*-/-*^ mice are highly susceptible to lethal influenza A virus infection with increasing pulmonary viral load, morbidity and mortality. Thus, our study highlight the importance of LGALS3BP in host antiviral innate immune responses.

## Introduction

The innate immune system senses a variety of pathogen-associated molecular patterns (PAMPs) via specific pattern-recognition receptors (PRRs), including the Toll-like receptor (TLR) and the RIG-I-like receptor (RLR)[1]. All TLRs, with the exception of TLR3, recruit myeloid differentiation primary response 88 (MyD88) to their receptor complex[2, 3]. The TLR/MyD88 complex in turn recruits members of the interleukin-1 (IL-1) receptor-associated kinase (IRAK) family. Activated IRAK family members dissociate from MyD88 and interact with tumor necrosis factor (TNF) receptor-associated factor 6 (TRAF6). TRAF6 forms a ubiquitin-conjugating enzyme complex that recruits transforming growth factor beta-activated kinase 1 (TAK1), leading to the I kappa B kinase (IKK) complex, composed of IKKα, IKKβ, and NEMO. As result, transcription factors, including nuclear factor-κB (NF-κB), interferon regulatory factor 3 (IRF3) and IRF7, translocate from the cytoplasm to nucleus[4, 5]. By contrast, TLR3 uses TIR domain-containing adaptor protein inducing IFNβ (TRIF) as the adapter protein. Activated TRIF recruits TRAF3, leading to TRAF family member-associated NF-κB activator (TANK) binding to TRAF3. Then, TANK recruits TBK-1 and/or IKKε to form a complex, causing IRF3 to translocate from the cytoplasm to the nucleus[6, 7]. After transcription factors migrate to the nucleus and assemble on the promoter, there is induction of type I interferons (IFN) and pro-inflammatory cytokines. These cytokines subsequently induce transcription of a wide range of antiviral and inflammatory genes that mediate innate antiviral immune and inflammatory responses[8, 9].

90K, also known as LGALS3BP, galectin-3-binding protein (Gal-3BP) and Mac-2-binding protein, is a ubiquitous multifunctional secreted glycoprotein originally studied in the context of neoplastic transformation and cancer progression[10, 11]. However, recently, several lines of evidence show that 90K expression is induced by various kinds of viral infections, including human immunodeficiency virus (HIV), hepatitis B virus (HBV), hepatitis C virus (HCV), hantavirus and dengue virus[12, 13]. Furthermore, 90K contains a scavenger receptor cysteine-rich (SRCR) domain, a BTB/POZ domain, a BACK (BTB and C-terminal Kelch) domain and an approximately 200 amino acid (aa) C-terminus with no significant similarity to other human proteins[14]. The SRCR domain is found in soluble or membrane-associated innate immunity-related proteins[15]. Many studies proposed intracellular and extracellular innate immunity functions for 90K. For example, 90K reduced the secretion of IL-4, IL-5, and IL-13 in PBMCs[16]; 90K treatment lead to CD14+ cell (monocytes/macrophages) induction by IL-1, IL-6 and TNF-α[17]; exogenous Gal-3BP has also been shown to activate THP-1 cells alone and additively with IFN-γ[18]. Although several functions of 90K in cytokine expression have been described, a clear role for 90K in innate immune responses to viral infections has not been established.

In this study, we show that many viruses, including influenza A virus (IAV), vesicular stomatitis virus (VSV) and herpes simplex virus (HSV) induce 90K expression. In turn, 90K limits viral expression and replication by inducing IFN and pro-inflammatory cytokines. Further experiments demonstrate that 90K functions as a scaffold protein to interact with TRAF6, TRAF3, TAK1 and TBK1, potentiates TRAF6 and TRAF3 ubiquitination and serves as a specific ubiquitination substrate of TRAF6. Taken together, our results suggest a novel mechanism for induction of IFN and pro-inflammatory cytokines during viral infections.

## Results

### Viral infection induces 90K expression

A previous study compared global gene expression differences in the liver in wild-type and HCV-infected chimpanzees by DNA microarray analysis[19, 20]. They found a number of gene expressions were changed during HCV infection. In this study, we investigated whether those genes affected HBV replication. As shown in S1 Fig, 90K and MAVS significantly inhibited the expression of HBeAg. Because the relationship between MAVS and virus has been widely reported, 90K was chosen for subsequent experiments.

**Fig 1:**
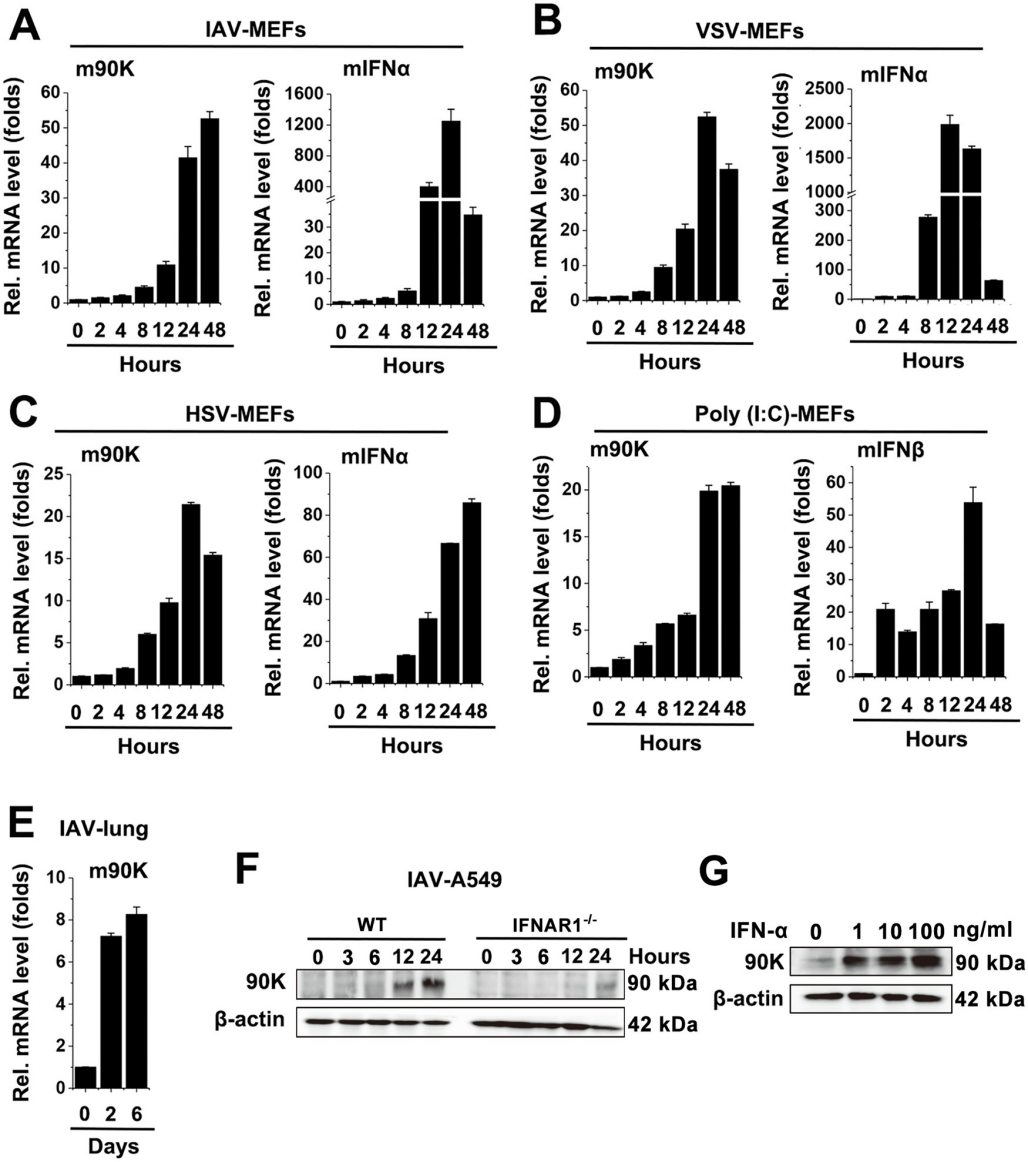
90K is induced by viruses. (A-C) C57BL/6 mice MEFs were infected with IAV (MOI = 1) (A), VSV (MOI = 1) (B), HSV-1 (MOI = 1) (C) for indicated times. 90K (left panel) and IFN-α (right panel) expression was quantified by real-time RT-PCR. (D) Experiments were performed as in (A), except that poly (I:C) was used. (E) C57BL/6 mice were infected with IAV (10^4^ TCID_50_ per mouse, n = 3) for indicated times. 90K mRNA levels in the lung were quantified by real-time RT-PCR. (F) A549 WT cells or IFNAR1^-/-^ cells were infected with IAV (MOI = 1) for indicated times prior to western blot assays. (G) A549 cells were treated with indicated concentrations of IFN-α for 24 hours. The protein level of 90K was analyzed by western blot. All experiments were repeated at least three times with consistent results. In the real-time RT-PCR experiments, the control was designated as 1. Bar graphs present means ± SD, n = 3 (**P < 0.01; *P < 0.05), n.s., not significant.

Since 90K was found elevated in patients infected with HIV, HBV, HCV, hantavirus and dengue virus, we determined whether other virus induce 90K expression. Results showed that influenza A virus (IAV), vesicular stomatitis virus (VSV) and herpes simplex virus (HSV) infection upregulated mRNA expression of 90K in mouse embryonic fibroblasts (MEFs) (Fig 1A–1C). IFN-α detection was included as a positive control for comparison (Fig 1A–1C). Similarly, poly(I:C), a synthetic dsRNA analog, also induced 90K expression (Fig 1D). 90K induction was also observed in IAV-infected murine lungs (Fig 1E). We next generated IFNAR1 knockout (IFNAR1^-/-^) cells to investigate the role of virus on 90K expression. As shown in Fig 1F, the protein levels of 90K were significantly reduced in IFNAR1^-/-^ cells compared with wildtype (WT) cells. Interestingly, IFN-α also induced 90K expression in A549 cells (Fig 1G). These data together suggested that these viral infections induce upregulation of 90K.

### 90K constrains viral replication *in vitro* and *in vivo*

**Fig 2:**
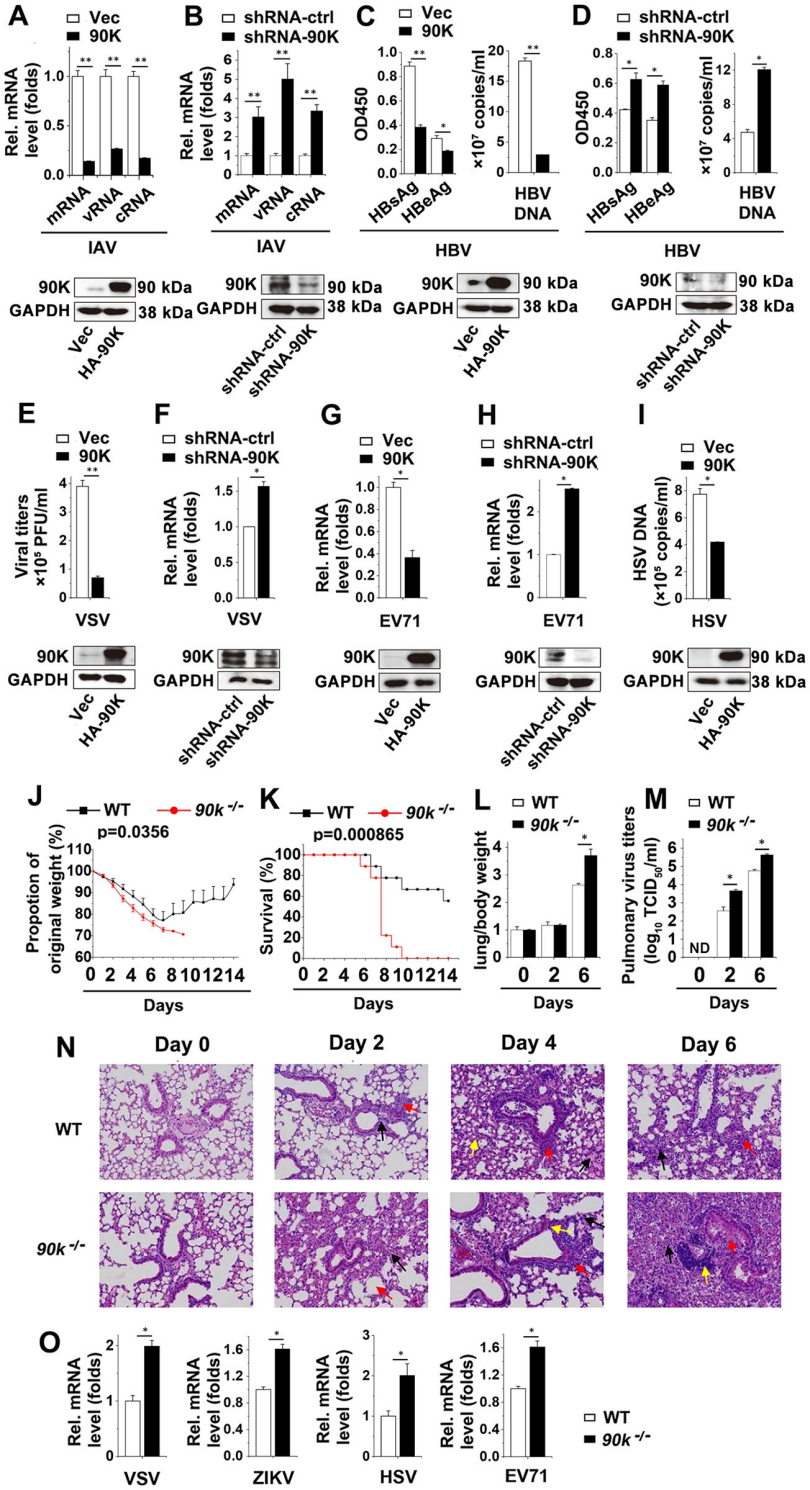
90K inhibits virus replication. (A) A549 cells were transfected with indicated plasmids for 24 hours followed by infection with IAV (MOI = 1) for 24 hours. The relative levels of NP-specific mRNA, cRNA and vRNA were quantified by real-time RT-PCR assay (upper panel) and protein levels of 90K were quantified by western blot (lower panel). (B) Experiments were performed as in (A) except cells were transfected with indicated shRNAs. (C) Huh7 cells were transfected with pHBV-1.3 and an empty vector or HA-90K for 48 hours. The secretion of HBeAg and HBsAg was measured by ELISA (left panel) and the amount of HBV capsid-associated DNA (right panel) was determined by real-time RT-PCR assay. (D) Experiments were performed as in (C) except cells were transfected with indicated shRNAs. (E) A549 cells were transfected with indicated plasmids for 24 hours followed by infection with VSV (MOI = 1) for 24 hours prior to plaque assay (upper panel). The protein levels of 90K were quantified by western blot (lower panel) (F) A549 cells were transfected with indicated shRNAs for 24 hours followed by infection with VSV (MOI = 1) for 8 hours prior to real-time RT-PCR assay (upper panel). The protein levels of 90K were quantified by western blot (lower panel). (G) RD cells were transfected with indicated plasmids for 24 hours followed by infection with EV71 (MOI = 1) for 2 hours prior to real-time RT-PCR assay. (H) Experiments were performed as in (G) except cells were transfected with indicated shRNAs. (I) Hela cells transfected with indicated plasmids for 24 hours followed by infection with HSV-1 for 24 hours prior to real-time RT-PCR assay. (J-M) WT (black square, n = 9) and *90k*^*-/-*^ (red circle, n = 9) mice were intranasally infected with 10^4^ TCID_50_ of IAV, and body weights were recorded daily (J). Survival curves show data collected until day 14 post-infection (K). The statistical analysis was performed using a log-rank test. Lung/body weight normalized (L) and viral titers (M) in lung tissues were evaluated on indicated times after influenza viral infection. (N) Histological analysis of the lung tissue of WT (n = 3) and *90k*^*-/-*^ mice (n = 3) stained with H&E on days 0, 2, 4, and 6 after intranasal infection with 10^4^ TCID_50_ IAV. (O) WT and *90k*^*-/-*^ splenocytes were infected with VSV (MOI = 1), ZIKV (MOI = 1), HSV (MOI = 1) or EV71 (MOI = 1) for 12 hours, respectively. The relative levels of nucleocapsid protein (VSV), envelope protein (ZIKV), ICP0 (HSV) or VP1 (EV71) were quantified by real-time RT-PCR assay. All experiments were repeated at least three times with consistent results. In the real-time RT-PCR experiments, the control was designated as 1. Bar graphs present means ± SD, n = 3 (**P < 0.01; *P < 0.05), n.s., not significant.

The finding that 90K was induced by various virus prompted us to investigate whether 90K influenced universal cellular antiviral activity. We first assessed the effect of 90K on IAV replication by measuring the production of three different forms of IAV RNA (mRNA, cRNA, and vRNA) using an approach described previously. Real-time RT PCR analyses showed that the levels of NP-specific mRNA, complementary RNA (cRNA) and vRNA were suppressed by 90K overexpression (Fig 2A). To confirm the effects of 90K on viral replication, we designed three specific shRNAs for 90K (shRNA-90K #1, #2, and #3) and tested their efficiency as shown in S2A Fig. ShRNA-90K #2 was selected for the experiments described below, in which we found that 90K knockdown increased IAV transcription and replication (Fig 2B). We next assessed the effects of 90K on HBV replication. The results indicated that 90K overexpression reduced HBeAg/HBsAg expression and HBV DNA replication, whereas 90K knockdown induced HBeAg/HBsAg expression and HBV DNA replication (Fig 2C and 2D). Furthermore, the effect of 90K on VSV production was also evaluated. As expected, VSV viral titers were significantly lower in 90K-overexpressing cells and VSV RNA levels were augmented when 90K was knocked down (Fig 2E and 2F). Similar results were also obtained in VSV-GFP infected cells by using flowcytometry and fluorescence microscopy (S2B and S2C Fig). We also investigated the effect of 90K on EV71 replication in RD cells. 90K overexpression effectively suppressed viral VP1 mRNA (Fig 2G) and protein (S2D Fig) levels. Conversely, 90K knockdown enhanced VP1 mRNA levels (Fig 2H). Similar results were also obtained in HSV-infected Hela cells (Fig 2I).

Mice deficient in the *90k* gene (*90k*^*-/-*^ mice) generated by standard knock-out embryonic stem cell system technology[21], with schematic diagram shown as S2E Fig, were used to further confirm the role of 90K in viral replication in *vivo*. We infected WT or *90k*^*-/-*^ mice with the A/FM/1/47 (H1N1) strain of influenza virus and monitored body weights. The *90k*^*-/-*^ mice exhibited lower body weights than WT mice during IAV infection (Fig 2J). Moreover, in response to the IAV infection, *90k*^*-/-*^ mice displayed an abbreviated survival time and lower survival rate than those of WT mice (Fig 2K). This increase in mortality was associated with higher lung/body index and significantly increased pulmonary viral load (Fig 2L and 2M). In line with the increasing in viral loads in *90k*^*-/-*^ mice, hematoxylin-and-eosin (H&E) staining showed greater infiltration of immune cells and damage in the lungs of *90k*^*-/-*^ mice than in WT mice after infection with IAV at various time points (Fig 2N). The role of 90K on replication of other viruses was also investigated. As shown in Fig 2O and S2F Fig, the replication of VSV, Zika virus (ZIKV), HSV and EV71 was greater in the *90k*^*-/-*^ mice splenocytes or MEFs than in WT mice. These results suggest that 90K accelerates an extensive cellular antiviral response against viral replication.

### 90K plays an important role in virus-induced proinflammatory cytokines and chemokines expression *in vitro* and *in vivo*

**Fig 3:**
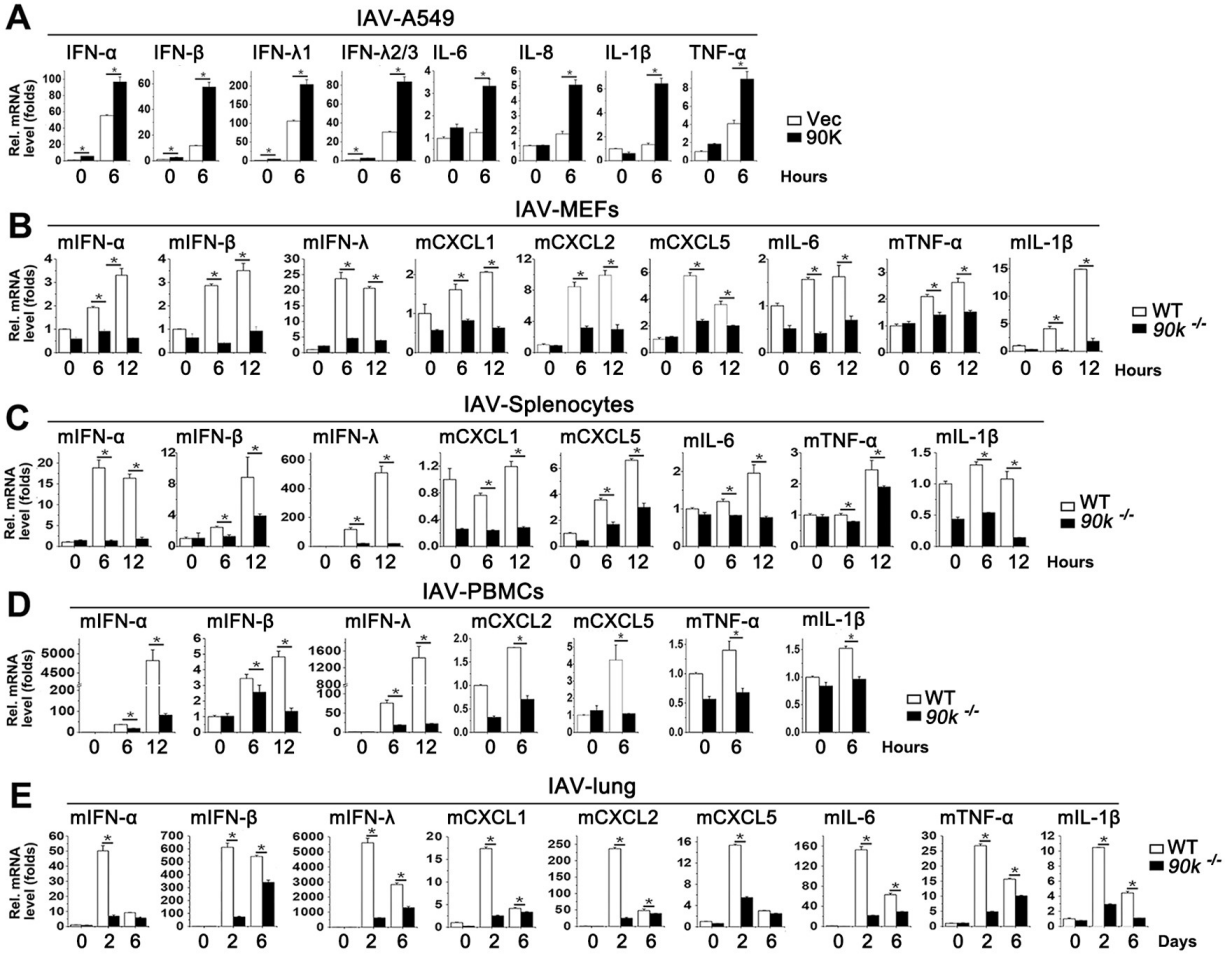
90K regulates IAV-induced inflammatory cytokine expression. (A) A549 cells were transfected with indicated plasmids for 24 hours and infected with IAV (MOI = 1) for indicated times prior to real-time RT-PCR assay. (B-D) qRT-PCR analysis of indicated cytokine mRNA in WT and *90k*^*-/-*^ MEFs (B), splenocytes (C), PBMCs (D) infected with IAV for indicated times. (E) WT (n = 3) and *90k*^*-/-*^ (n = 3) mice were intranasally infected with 10^4^ TCID_50_ of IAV for indicated times. The relative levels of inflammatory cytokine in mice lung were quantified by real-time RT-PCR assay. All experiments were repeated at least three times with consistent results. In the real-time RT-PCR experiments, the control was designated as 1. Bar graphs present means ± SD, n = 3 (**P < 0.01; *P < 0.05), n.s., not significant.

IFN and cytokines regulate a broad range of viral infections. Since 90K also affects replication of many viruses, we investigated whether 90K regulated IFN and pro-inflammatory production. Using luciferase activity reporter assays, we showed that 90K overexpression stimulated ISRE, NF-κB, IFN-β and IFN-λ promoter (S3A Fig). Elevated mRNA levels of IFN-α, IFN-β, IFN-λ1 and IFN-λ2/3 were also observed in HSV-infected HeLa cells when 90K was overexpressed (S3B Fig). Several lines of evidence support the notion that some kinds of IAV infection induce massive release of inflammatory cytokines and chemokines, called the ‘cytokine storm’. Thus, we examined the role of 90K in IAV-induced cytokine storm. As shown in Fig 3A, 90K overexpression enhanced IAV-induced IFN and pro-inflammatory production in A549 cells. We next investigated whether the production of proinflammatory cytokines and chemokines was altered in *90k*^*-/-*^ mice during the IAV infection. Real-time RT-PCR showed that the mRNAs levels of proinflammatory cytokines and chemokines were significantly lower in MEFs, splenocytes, PBMCs and lungs of *90k*^*-/-*^ mice than in WT mice during IAV infection (Fig 3B–3E). Similar results were also obtained in poly (I:C) treated A549 cells or *90k*^*-/-*^ mice and WT mice MEFs, splenocytes and PBMCs. (S3C–S3F Fig). We also examined the role of 90K on other virus-regulated cytokine expressions. Lower mRNA levels of cytokines were observed in MEFs and PMBCs of *90k*^*-/-*^ mice than in WT mice during VSV, EV71, ZIKV and HSV infections (S3G and S3H Fig). These data collectively indicate that 90K positively regulated virus-triggered proinflammatory cytokine and chemokine expression.

### 90K potentiates transcription factors translocation and ISG induction

**Fig 4:**
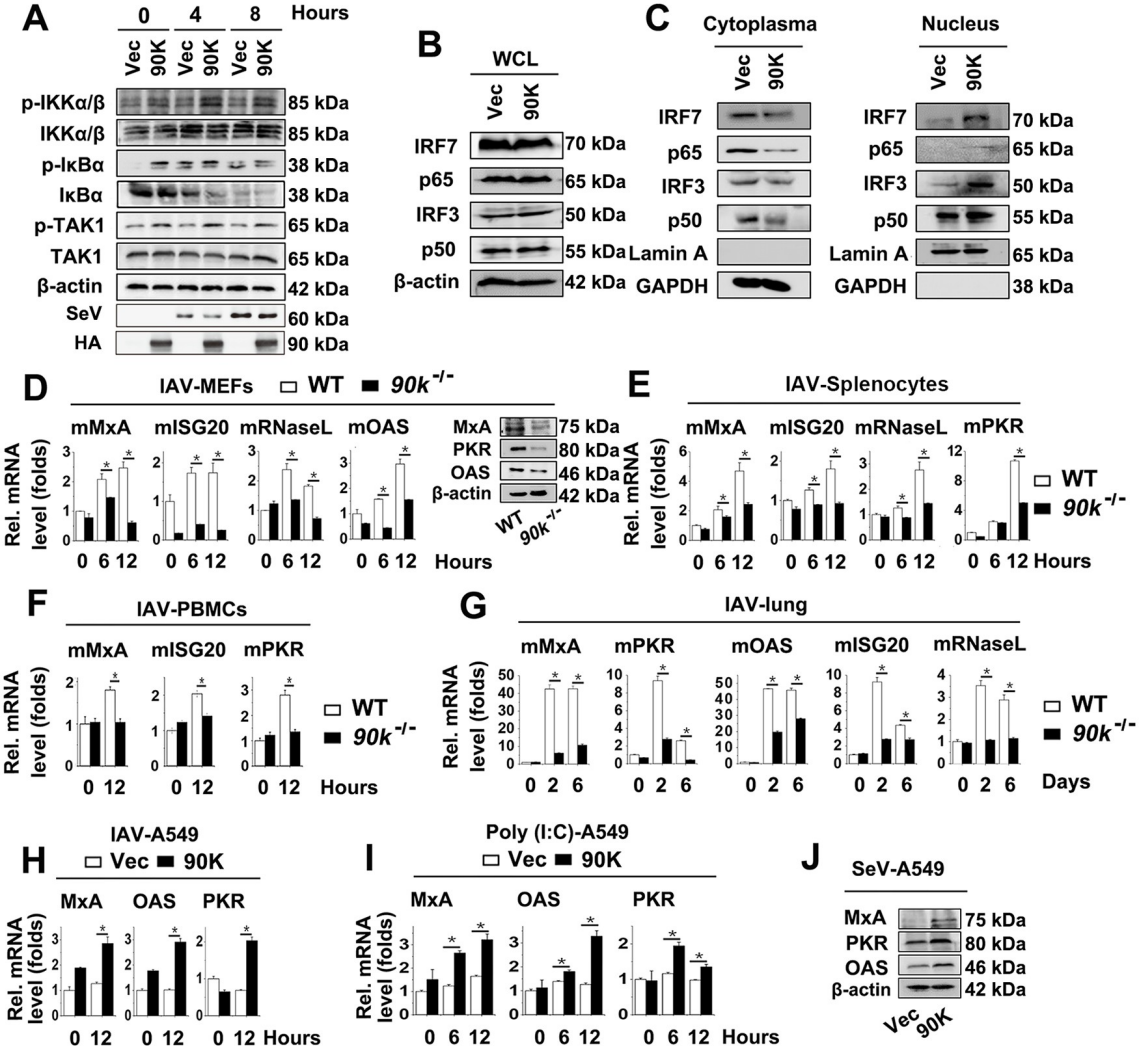
90K regulated IRF3, IRF7 and NF-κB activation and ISGs expression. (A) A549 cells infected with SeV for indicated times prior to western blot analyses. (B) A549 cells transfected with indicated plasmids for 24 hours prior to western blot analyses. (C) A549 cells transfected with indicated plasmids for 24 hours and infected with IAV (MOI = 1) for 6 hours. Cytosolic and nuclear extracts were prepared and subjected to western blot analyses. Lamin A and GAPDH were used as markers for nuclear and cytosolic fractions, respectively. (D-F) WT and *90k*^*-/-*^MEFs (D), splenocytes (E), PBMCs (F) were infected with IAV for indicated times. The relative levels of ISGs were quantified by real-time RT-PCR or Western blot analyses. (G) WT (n = 3) and *90k*^*-/-*^ (n = 3) mice were intranasally infected with 10^4^ TCID_50_ of IAV for indicated times. The relative levels of ISGs in mice lung were quantified by real-time RT-PCR. (H and I) A549 cells were transfected with indicated plasmids for 24 hours and infected with IAV (H) or treated with poly (I:C) (I) for indicated times. The relative levels of ISGs were quantified by real-time RT-PCR analyses. (J) A549 cells ere transfected with indicated plasmids for 24 hours and infected with SeV for 24 hours prior to western blot analyses. All experiments were repeated at least three times with consistent results. In the real-time RT-PCR experiments, the control was designated as 1. Bar graphs present means ± SD, n = 3 (**P < 0.01; *P < 0.05), n.s., not significant.

Because we found that 90K potentiated pro-inflammatory cytokine expression, we speculated that 90K was involved the upstream and downstream events of pro-inflammatory cytokine expression. To test this hypothesis, we investigated the effect of 90K on the IKK complex. Western blot experiments indicated that overexpression of 90K increased the phosphorylation of IκBα, IKKα/β and TAK1 (Fig 4A). Because induction of pro-inflammatory cytokines requires coordinated and cooperative actions of the transcription factors IRF3/7 and NF-κB, we wondered whether 90K was involved this process. In Western blot assays, 90K overexpression clearly enhanced the translocation of the NF-κB subunits p50/p65 as well as IRF3/7 from the cytosol to the nucleus after SeV infection, whereas total protein levels of p65/50 and IRF3/7 were not affected (Fig 4B and 4C). Similar results were obtained by immunofluorescence assays in A549 cells and MEFs (S4A and S4B Fig).

We further examined whether the expression of IFN-stimulated genes (ISGs) was affected by 90K. Results showed that ISG levels were significantly lower in the MEFs, splenocytes, PBMCs and lungs of *90k*^*-/-*^ mice than in WT mice during the IAV infection (Fig 4D–4G). Similar results were obtained of poly (I:C)-treated *90k*^*-/-*^ mice and WT mice MEFs, splenocytes and PBMCs. (S4C–S4E Fig). The role of 90K on ISG expression was also investigated *in vitro* using IAV, poly (I:C) and SeV. Consistently, overexpression of 90K enhanced viral- and poly (I:C)-induced ISG expression (Fig 4H–4J). These data collectively indicate that 90K positively regulates virus-triggered signaling and ISG expression.

### 90K interacts with TRAF6 and TAK1

**Fig 5:**
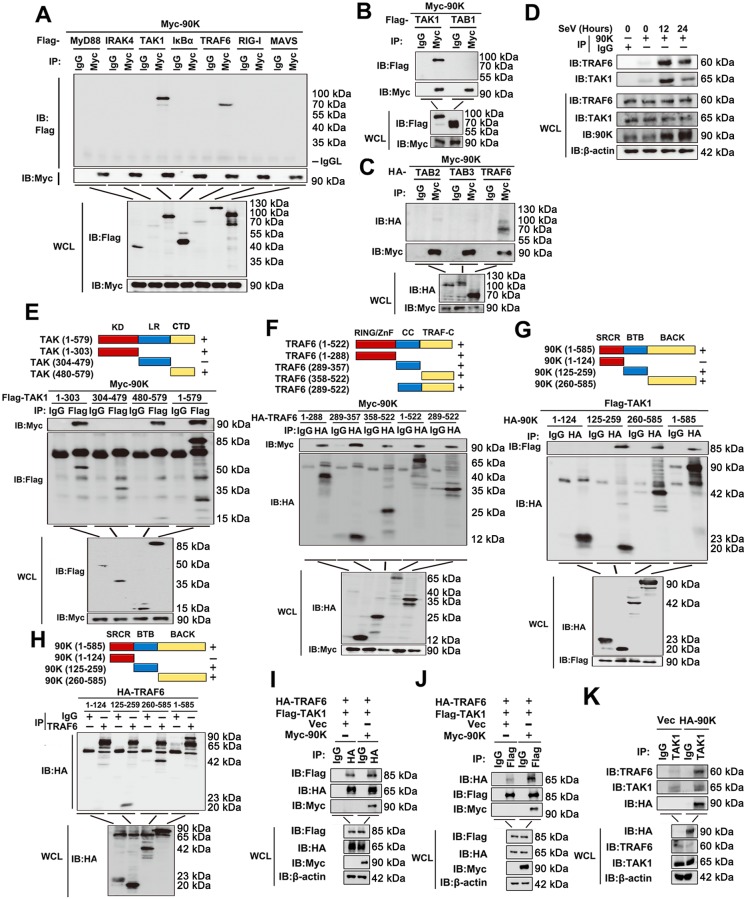
90K associates with TRAF6 and TAK1. (A-C) 293T cells were transfected with the indicated plasmids for 48 hours. Coimmunoprecipitation and immunoblots were performed with the indicated antibodies. (D) A549 cells were infected with SeV for the indicated times or left uninfected. Immunoprecipitation and immunoblot analysis were performed with the indicated antibodies. (E-H) Schematic diagram of the full-length and truncated constructs of TAK1, TRAF6 and 90K (upper panel). 293T cells were transfected with the indicated plasmids for 48 hours. (I-K) 293T cells were transfected with the indicated plasmids for 48 hours. Coimmunoprecipitation and immunoblots were performed with the indicated antibodies. All experiments were repeated at least three times with consistent results.

Because we found that 90K induced phosphorylation of IκBα, IKKα/β and TAK1, we speculated that 90K associated with some components upstream of the IKK complex. To test this hypothesis, we investigated the relationship between 90K and some complexes that lie upstream of the IKK complex. The results of transient transfection and co-immunoprecipitation (Co-IP) experiments indicated that Myc-tagged 90K interacted with Flag-tagged TRAF6 and TAK1, but not with other components, including MyD88, IRAK4, IκBα, RIG-I, MAVS, TAB1, TAB2 and TAB3 (Fig 5A–5C). Using HA-tagged 90K, we further confirmed the interaction of 90K with TRAF6 and TAK1 via Co-IP and reverse Co-IP experiments (S5A and S5B Fig). These results were further supported by confocal microscopy observations that 90K and TRAF6 and TAK1 co-localized in 293T and A549 cells during SeV infection (S5C–S5E Fig). We further performed endogenous Co-IP experiments, and the results indicated that 90K was weakly associated with TRAF6 and TAK1 in unstimulated cells, and this association increased after stimulation with SeV (Fig 5D). To map the region of 90K that interacted with TRAF6 and TAK1, we constructed a series of truncations of 90K, TRAF6 and TAK1 (Fig 5E–5H, upper panel). We further demonstrated that N-terminus kinase domain (aa 1–303) and C-terminus domain (aa 480–579) of TAK1 were required for its binding to 90K, while 90K interacted with all truncations of TRAF6 (Fig 5E and 5F). Next, we found that the BTB domain (aa 125–259) and BACK domain (aa 260–585) of 90K were required for association with both TRAF6 and TAK1 (Fig 5G and 5H).

Because 90K interacts with both TRAF6 and TAK1, we reasoned that 90K might enhance the interaction between TRAF6 and TAK1. Results from Co-IP and reverse Co-IP assays indicated that the interaction between TRAF6 and TAK1 was substantially enhanced by 90K (Fig 5I and 5J). We further performed endogenous Co-IP experiments, and the results indicated that VSV-induced TRAF6 and TAK1 association, and this association increased after transfected with 90K overexpression plasmid (Fig 5K). Collectively, these data indicate that 90K reinforces the recruitment of TAK1 to TRAF6.

### 90K potentiates TRAF6 auto-ubiquitination, and TRAF6 in turn ubiquitinates 90K

**Fig 6:**
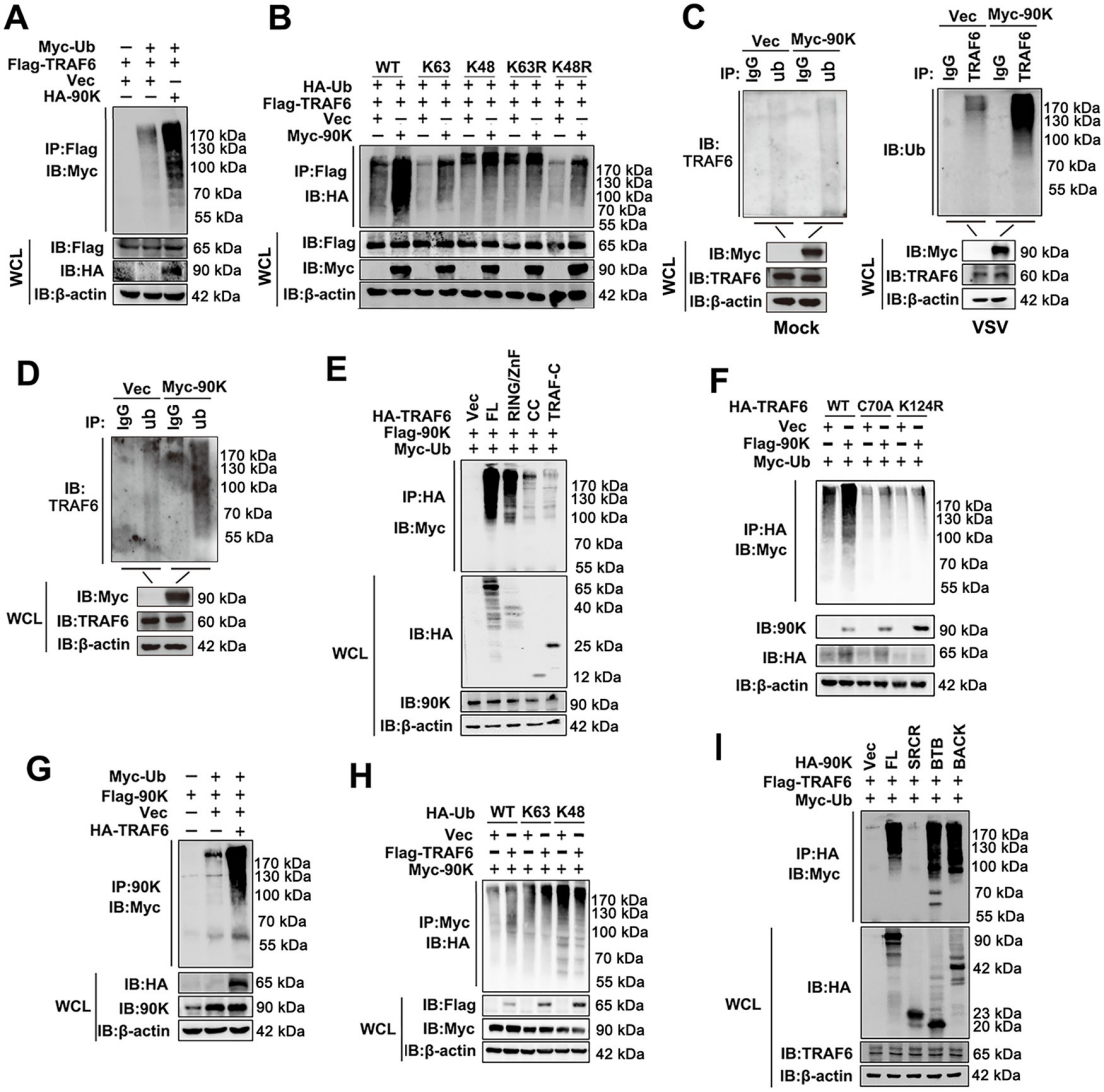
90K potentiates TRAF6 ubiquitination and is ubiquitinated by TRAF6. (A and B) 293T cells were transfected with HA or Myc tagged 90K plasmids and Flag-TRAF6 and the indicated ubiquitin plasmids for 48 hours before co-immunoprecipitation and immunoblot analyses were performed with the indicated antibodies. (C) 293T cells transfected with empty vector or Myc-90K for 24 hours. Cells were infected without (left panel) or with (right panel) VSV for 6 hours. Coimmunoprecipitation and immunoblot were performed with the indicated antibodies. (D) 293T cells transfected with empty vector or Myc-90K for 24 hours and infected with VSV for 6 hours. Coimmunoprecipitation and immunoblot were performed with the indicated antibodies. (E and F) 293T cells were transfected with Flag-90K and Myc-Ub or indicated truncated or mutated TRAF6 constructs for 48 hours. Coimmunoprecipitation and immunoblot were performed with the indicated antibodies. (G and H) 293T cells were transfected with the indicated plasmids for 48 hours before co-immunoprecipitation and immunoblot analyses were performed with the indicated antibodies. (I) 293T cells were transfected with Flag-TRAF6 and Myc-Ub or indicated truncated 90K constructs for 48 hours. Coimmunoprecipitation and immunoblot were performed with the indicated antibodies. All experiments were repeated at least three times with consistent results.

TRAF6 has been shown to undergo lysine-63 (K63)-linked auto-ubiquitination. Because we found that 90K interacts with TRAF6, we speculated that 90K regulates TRAF6 ubiquitination. We performed ubiquitination assays to determine whether 90K altered TRAF6 polyubiquitination. Co-expression of 90K in the presence of exogenous ubiquitin clearly accelerated TRAF6 ubiquitination (Fig 6A). Another experiment suggested that 90K enhanced K63-linked, but not K48-linked, polyubiquitination of TRAF6 (Fig 6B). Consistently, knockdown of 90K did not affect the expression of TRAF6 and TAK1 (S5F Fig). We next assessed the levels of endogenous TRAF6 polyubiquitination in 90K-overexpressing 293T cells infected with VSV. Results showed that significantly higher levels of TRAF6 polyubiquitination were obtained in the presence of 90K in VSV infected cells (Fig 6C). Similar results were also obtained using ubiquitin antibody for immunoprecipitation and TRAF6 antibody for immunoblot (Fig 6D). TRAF6 consists of an N-terminal RING finger domain, a series of four internal zinc finger motifs, an α-helical coiled-coil domain, and a C-terminal TRAF-C domain. We next investigated which domain of TRAF6 underwent polyubiquitination enhanced by 90K. Notably, 90K accelerated polyubiquitination of full-length and the RING/ZnF domain of TRAF6 (Fig 6E). It has been reported that the TRAF6-C70A mutation in the RING domain abolishes the ligase activity of TRAF6 and Lys-124 is the predominant ub acceptor site for TRAF6-mediated auto-ubiquitination[22, 23]. We next investigated the role of those two residues in 90K regulated TRAF6 ubiquitination. Results showed that 90K failed to induce polyubiquitination of TRAF6 C70A and K124R mutants (Fig 6F).

Because TRAF6 also facilitates a number of signaling pathways by catalyzing K63-linked ubiquitination of specific substrates, we speculated that 90K may be a specific substrate of TRAF6 involved in the anti-viral signaling pathway. In an overexpression system, TRAF6 enhanced polyubiquitination of 90K (Fig 6G). Further study indicated that TRAF6 enhanced K63-linked, but not K48-linked polyubiquitination of 90K (Fig 6H). We next sought to determine which domain of 90K undergoes polyubiquitination. As shown in Fig 6I, TRAF6 induced polyubiquitination of the BTB and BACK domains, but not the SRCR domain. We next determined the antiviral activity of these three mutants. Consistently, the BTB and BACK domains of TRAF6 inhibited IAV replication (S6 Fig). In contrast, the SRCR domain of TRAF6 did not affect IAV replication (S6 Fig). These results suggest that 90K, a substrate of TRAF6, promotes TRAF6 ubiquitination.

### 90K interacts with TRAF3 and TBK1 and potentiates TRAF3 ubiquitination

**Fig 7:**
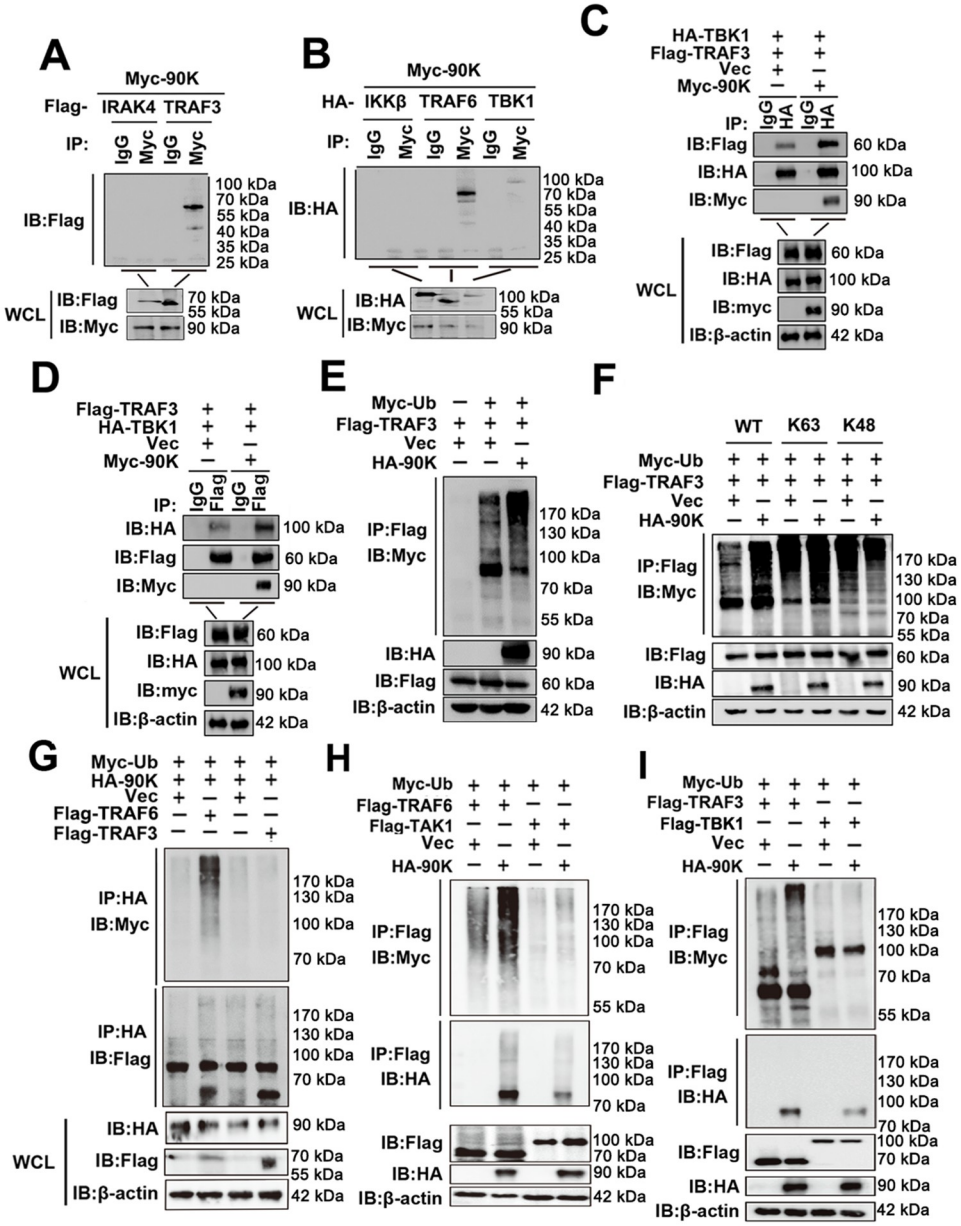
90K interacts with TRAF3 and TBK1, and enhances TRAF3 ubiquitination. (A and B) 293T cells were transfected with the indicated plasmids for 48 hours. Coimmunoprecipitation and immunoblots were performed with the indicated antibodies. (C and D) 293T cells were transfected with the indicated plasmids for 48 hours. Coimmunoprecipitation and immunoblots were performed with the indicated antibodies. (E-I) 293T cells were transfected with the indicated plasmids and Myc-Ub for 48 hours. Coimmunoprecipitation and immunoblots were performed with the indicated antibodies. All experiments were repeated at least three times with consistent results.

Given that TRAF3 also plays an important role in TLR an RLR signaling, we also investigated the role of 90K in TRAF3-mediated signaling. Co-IP experiments indicated that Myc-tagged 90K interacted with Flag-tagged TRAF3 and HA-tagged TBK1 (Fig 7A and 7B). And the interaction between TRAF3 and TBK1 was substantially enhanced by 90K (Fig 7C and 7D). Further study indicated that 90K elevated polyubiquitination of TRAF3 (Fig 7E). Furthermore, overexpression of 90K potently promoted K63-linked polyubiquitination of TRAF3 but had only a minimal effect on K48-linked polyubiquitination of TRAF3 (Fig 7F). Different from TRAF6, 90K didn’t serve as a specific ubiquitination substrate of TRAF3 (Fig 7G). Interestingly, 90K failed to mediate polyubiquitination of TAK1 and TBK1 (Fig 7H and 7I). These results suggest that 90K associates with TRAF3 and TBK1, reinforces the recruitment of TBK1 to TRAF3 and mediates K63-linked polyubiquitination of endogenous TRAF3.

## Discussion

We identified a previously-unrecognized role for inducible 90K in IFN and pro-inflammatory cytokine production in response to viral infections. 90K exhibited strong antiviral activity toward a broad range of viral infections. Our study provided several lines of evidence that 90K is a novel activator of NF-κB and IRF3/7 signaling by positive regulation of TRAF6 and TRAF3 function.

Currently, several reports have shown that both chronic and acute viral infections induce 90K expression. One group observed that high 90K concentrations in HIV-infected individuals were associated with faster progression towards acquired immune deficiency syndrome (AIDS)[24]. Other investigators observed high levels of 90K expression in the serum of HBV and HCV patients, and correlated with the severity of liver damage[25]. Similar results were also observed in acute viral infections. DENV, hantavirus also induced 90K expression[12, 26]. In the current study, we investigated the relationship between 90K and other three viruses, including IAV, VSV and HSV. These viruses also induced 90K expression. Intriguingly, 90K inhibits replication of many kinds of viruses, including IAV, HBV, ZIKV, EV71, VSV and HSV. Further studies demonstrated that it plays an important role in virus-induced IFN and pro-inflammatory cytokine production *in vitro* and *in vivo*. In light of previous studies and our current results, we propose that 90K is a novel virus-induced host factor that exhibits antiviral activity toward a broad range of viral infections. This character of 90K is similar to that of major vault protein (MVP), a novel virus-induced host factor identified by our team recently that also inhibited replication of many viruses by up-regulating type-I interferon production[27].

Scaffold proteins interact and/or bind with several members of a signaling pathway, tethering them into complexes. Adaptor proteins are accessories to main proteins in a signal transduction pathway. Adaptor proteins include a variety of protein-binding modules that link protein-binding partners together and facilitate the creation of larger signaling complexes. In innate immunity, there are some well-known scaffold/adaptor proteins. For example, MyD88 and TRIF are crucial adaptor proteins that lie downstream of TLRs[28]. Those adaptor proteins interact with other signaling molecules, including members of the IRAK and TRAF families. TRAF6 is essential for the activation of most known MyD88-mediated effector pathways, but are dispensable for TRIF-dependent NF-κB, IRF3 and IRF7 activation[29]. TRIF-dependent NF-κB activation is still not fully understood. Several studies confirmed that the adaptor protein TNFR1-associated death domain protein (TRADD) and the serine/threonine kinase receptor-interacting protein 1 (RIP1) play important roles in TRIF-mediated NF-κB activation[30, 31]. On the other hand, TRAF3 is recruited to both the MyD88- and TRIF-assembled signaling complexes and positively control IRF3 and IRF7 activation[7, 32]. In this study, we further investigated the mechanisms of 90K in cellular antiviral responses. To our surprise, 90K interacts with both TRAF6 and TAK1, but not with MyD88 and IRAKs, and promotes the association between TRAF6 and TAK1. Therefore, we believe that 90K may function as a scaffold/adaptor protein, downstream of MyD88 and IRAKs, connecting TRAF6 and TAK1 interaction. Interestingly, in this study, we also observed the interaction between 90K and TRAF3 and TBK1, indicating that 90K may function as another candidate for a scaffold/adaptor protein connecting TRAF3 and TBK1. Further study verified that 90K potentiates NF-κB, IRF3 and IRF7 translocation. These results provide a detailed understanding of TRIF-dependent NF-κB activation.

Apart from their role as adaptor proteins, TRAF proteins also act as E3 ubiquitin ligases, a function that is crucial for the activation of downstream signaling events[33, 34]. In this study, we showed that 90K is a specific substrate of TRAF6. Moreover, 90K possesses a BTB domain that defines a recognition motif for the assembly of substrate-specific RING/cullin 3/BTB ubiquitin ligase complexes[35]. We suspect that 90K may affect TRAF6 and TRAF3 ubiquitination. Interesting, 90K not only interacts TRAF6 and TRAF3, but also promotes TRAF6 and TRAF3 ubiquitination. Nevertheless, we still do not know whether 90K is an E3 ubiquitin ligase or whether 90K recruits E3 ubiquitin ligases to TRAF6 and TRAF3. When considering the next step, studies exploring these questions would be of great help in further clarifying the role of 90K in innate immunity.

**Fig 8:**
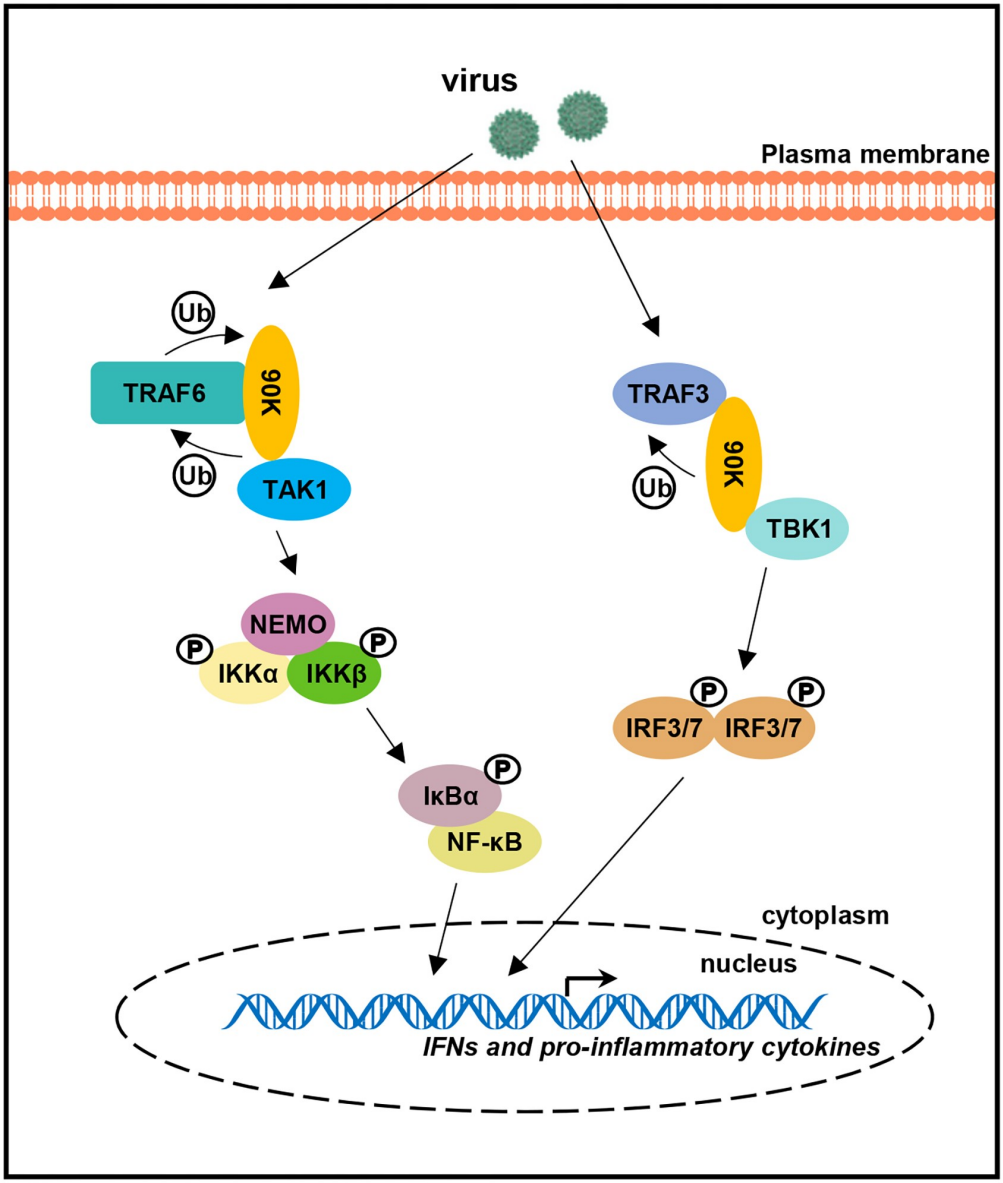
Model of the biological effect of 90K in virus-induced inflammatory cytokine expression. After virus infection, TRAF6 recruits the newly-expressed 90K through its RING/ZnF domain. Association of the TRAF6 and 90K forms a ubiquitin-conjugating enzyme complex that recruits TAK1. Induced 90K also interacts with TRAF3 and TBK1 to form a complex. Two complexes signal to translocate the transcription factors IRF3/7 and NF-κB, from the cytoplasm to the nucleus for subsequent production of IFN and inflammatory cytokines.

We propose a working model describing the role of 90K in innate immunity regulated by virus (Fig 8). In this model, viral infection strongly induces 90K expression. Subsequently, 90K interacts with TRAF6 or TRAF3, leading to TRAF6 or TRAF3 ubiquitination. This complex in turn recruits TAK1 and TBK1, that signals to translocate the transcription factors NF-κB, IRF3 and IRF7,from the cytoplasm to the nucleus for subsequent production of IFN and inflammatory cytokines. In conclusion, the results of this study reveal a previously-undescribed role for 90K in regulating viral replication and antiviral responses to advance our knowledge of the host immune response to viral infection.

## Materials and methods

### Ethics statement

All animal experiments in this study were in accordance with protocols and procedures approved by the Institutional Animal Care and Use Committee of Wuhan University (approval number WDSKY0200902-2). The animal care and use protocol was adhered to the Chinese National Laboratory Animal-Guideline for Ethical Review of Animal Welfare.

### Experimental animals and *in vivo* virus infection

The C57BL/6 90K^-/-^ mice were kindly provided by Prof. Klaus Ley (La Jolla Institute for Allergy & Immunology, La Jolla, CA). Age- and sex-matched wild-type C57BL/6 mice were purchased from the Center for Animal Experiment of Wuhan University. All mice were housed in the specific pathogen-free animal facility at Wuhan University.

8–12-week-old mice were infected with mouse-adapted influenza virus A/FM/1/47 (H1N1) with 1000-fold tissue culture-infective dose at 50% (TCID_50_) per mouse by intranasal instillation. Body weights and survival were monitored for 14 days. Pulmonary virus quantitation was previously described[36]. Briefly, mice were euthanized and dissected; mice lungs were homogenized in influenza virus growth medium (DMEM supplemented with 0.2% BSA solution, 100 U/ml penicillin, 100 mg/ml streptomycin, 2 mM L-glutamine, 25 mM HEPES buffer, 2 mg/ml TPCK trypsin) on days 0, 2, and 6 post-infection, and lung viral titers were determined by a modified TCID_50_ assay reported by the World Health Organization and calculated according to the Reed-Muench method. Mice lungs were fixed in 10% neutral buffered formalin, embedded in paraffin, sectioned, and stained with hematoxylin-eosin and examined by light microscopy at 200× magnification for histological changes. For measurement of lung/body index, mice were euthanized and the body and lung tissues were weighed on days 0, 2, and 6 post-infection and the corresponding ratio was calculated. For cytokine studies, mice were sacrificed and lungs were harvested for qRT-PCR at day 0, 2 and 6 after infection.

### Preparations of MEFs, splenocytes, and PBMCs

MEFs were prepared from day 13.5 C57BL/6 mice embryos and cultured in Dulbecco’s modified Eagle’s medium (DMEM) supplemented with 10% fetal bovine serum 1% penicillin-streptomycin. Murine PBMCs were isolated from blood using mouse lymphocyte separation medium (TBD, Tianjin, China) and cultured in RPMI 1640. Murine splenocytes were obtained by processing spleens through a disposable cell strainer and erythrocytes were removed by incubation in hypotone Tris/NH_4_Cl red cell lysis buffer and were cultured in endotoxin-free DMEM.

### Antibodies and reagents

Antibodies against Flag (M185-3L), HA (M180-3) and Myc (M192-3) were purchased from Medical & Biological Laboratories. Antibodies against TRAF6 (sc-8409), PKR (sc-100378) and OAS (sc-98424) were purchased from Santa Cruz Biotechnology. Antibodies against 90K (60066-1-Ig, 10281-1-AP), TAK1 (12330-2-AP), MxA (13750-1-AP), IRF3 (11312-1-AP), NF-κB p65 (10745-1-AP), NF-κB p50 (14220-1-AP), lamin A/C (10298-1-AP), β-actin (60008-1-Ig), GAPDH (60004-1-Ig) and β-tubulin (66249-1-Ig) were purchased from ProteinTech Group. Antibodies against p-TBK1 (5483), p-IRF3 (4947), p-IKK (2697S), IKK (2678), p-IκBα (2859S), IκBα (4812S) were purchased from Cell Signaling Technology. Antibodies against TBK1 (ab40676) was purchased from Abcam. Antibodies against EV71 VP1 (PAB7631-D01P) was purchased from Abnova. Poly(I:C) were purchased from InvivoGen.

### RNA interference

Double-stranded oligonucleotides targeting to 90K sequences were cloned into the pLKO.1 puro cloning vector with shRNA construct (Addgene) as previously described. The target sequences designed for 90K mRNA as follows:

#1: 5’-GAGCGCTCAGCTTCAAGAAAT-3’,

#2: 5’-TGTGGTCTGCACCAATGAAAC-3’,

#3: 5’-ATCGCACCATTGCCTACGAAA-3’.

### Generation of IFNAR1 knockout cell line

A549 IFNAR1 KO cell line was generated by CRISPR-Cas9 system described before[37]. The following oligonucleotides specific targeting the IFNAR1 gene were used:

oligonucleotide 1, 5’-CACCGACCCTAGTGCTCGTCGCCG-3’;

oligonucleotide 2, 5’-AAACCGGCGACGAGCACTAGGGTC-3’.

### Measurement of IAV replication

IAV replication was determined as previously described with modification[37]. A549 cells (obtained from China Center for Type Culture Collection) were infected with IAV/Hong Kong/498/97 (H3N2) at a multiplicity of infection (MOI) of 1 for 24 h. Cells were harvested and total RNA was extracted with TRIzol according to the manufacturer’s instructions. The relative RNA levels of nucleoprotein (NP)-mRNA, NP-cRNA, and NP-vRNA were determined using quantitative real-time RT-PCR (qRT-PCR) as previously described. The following primers were used for the reverse transcription: NP-vRNA, 5’-CTCACCGAGTGACATCAACATCATG-3’; NP-cRNA, 5’-AGTAGAAACAAGGGTA-3’; and NP-mRNA, oligo(dT). The following primers were used for qRT-PCR: NP, 5’-ATCAGACCGAACGAGAATCCAGC-3’ (sense) and 5’-GGAGGCCCTCTGTTGATTAGTGT-3’ (antisense).

### Purification and quantification of HBV capsid-associated DNA

Capsid-associated DNA was extracted as described previously[38], with modifications. At 72 h post-transfection of indicated plasmids together with pHBV1.3, Huh7 cells (obtained from China Center for Type Culture Collection) were lysed in 1 ml of lysis buffer 50 mM Tris, pH 7.5, 0.5% NP-40, 1 mM EDTA, and 100 mM NaCl) and mixed gently at 4 °C for 1 h. and subsequently incubated with 10 μl of 1 M MgCl_2_ and 10 μl of DNase I (10 mg/ml, Takara) at 37 °C for 2 h. DNA that was not protected by HBV core protein was digested with DNase I. Viral cores were then precipitated by adding 35 μl (0.5 M) of EDTA and 225 μl of 35% polyethylene glycol and incubating them at 4 °C for at least 30 min, after which the cores were concentrated by centrifugation and the pellets were resuspended in 10 mM Tris, 100 mM NaCl, 1 mM EDTA, 1% sodium dodecyl sulfate (SDS), and 20 μl of proteinase K (25 mg/ml) and incubated overnight. Viral DNAs released from lysed cores were extracted with phenol and chloroform, precipitated with isopropanol and resuspended in Tris-EDTA. Absolute quantitative RT-PCR was performed using pHBV1.3 dilutions as standards with a set of primers for HBV DNA detection as follows: 5′-AGAAACAACACATAGCGCCTCAT-3′ (sense), 5′-TGCCCCATGCTGTAGATCTTG-3′ (antisense).

### VSV plaque assays

A549 cells cultured in 12-well plates were transfected with the indicated plasmids for 24 hours followed by infection with VSV-GFP (MOI of 1). After one hour, the cells were washed with warm PBS and fresh medium was added. Twelve hours later, the supernatants were collected and diluted to 10^−6^, 10^−5^, 10^−4^, 10^−3^ and 10^−2^ with DMEM and used to infect confluent Vero cells (obtained from China Center for Type Culture Collection) cultured in 24-well plates. One hour later, the cells were washed with PBS twice and cultured in a mixture of warm 3% low-melting point agarose and DMEM containing 10% FBS, 1% methylcellulose and 1% streptomycin and penicillin for 72 hours. Cells were stained with 0.2% crystal violet for 2 hours, and the overlays were removed. The numbers of plaques were counted, averaged and multiplied by the dilution factor to determine the viral titer (PFU/ml).

### Luciferase assays

A549 cells were seeded in 24-well plates and transfected with the indicated plasmid and luciferase reporter plasmid at an appropriate ratio together with pRL-TK (Renilla luciferase plasmid) as internal control and empty vector was used to equalize the total amount of DNA. Cells were disrupted with lysis buffer (Promega), and luciferase activity was measured by the Dual-Luciferase Reporter Assay (Promega) 24 hours post-transfection according to the manufacturer’s instructions. The firefly luciferase enzyme activity was normalized to Renilla luciferase enzyme activity and expressed as fold-expression.

### Preparation of nuclear and cytoplasmic extracts

Cells were washed with ice-cold PBS, collected by centrifugation, and resuspended in hypotonic cytoplasm lysis buffer (10 mM Tris-HCl [pH 7.4], 5 mM MgCl_2_, 10 mM NaCl, 1 mM DTT, 10% protease inhibitor mixture) for 15 min on ice prior to incubation with 0.5% Nonidet P-40 on ice for 1 min. Cell lysates were centrifuged at 13,000 g for 10 min, and the supernatants were collected as cytoplasm extractions. The pellets were washed once and resuspended in nucleus lysis buffer (20 mM HEPES-KOH [pH 7.9], 1.5 mM MgCl_2_, 0.5 mM NaCl, 1 mM DTT, 0.2 mM EDTA, 10% protease inhibitor mixture, 1% Nonidet P-40) for 30 min on ice. Nuclear extractions were harvested by centrifugation at 13,000 g for 10 min. All fractions were snap-frozen in liquid nitrogen and stored at -70 °C until use.

### Quantitative real-time PCR and ELISA

Total RNA was isolated with the TRIzol reagent (Invitrogen) and cDNA was synthesized using the TRUEscript H Minus M-MuLV Reverse Transcriptase (Aidlab Biotechnologies). Data shown are the relative abundances of the indicated mRNA derived from human or mouse cells normalized to those of GAPDH or HPRT, respectively. Gene expression was examined with a Bio-Rad CFX connect system with iTaq Universal SYBR Green Supermix (Biorad). Gene-specific primer sequences were as described or reported in S1 Table. Protein levels of HBV e/s antigen in supernatants were measured by ELISA kit (Shanghai Kehua Bio).

For HSV DNA qualification, cells were lysed and DNA was isolated using genomic DNA extraction kits (Aidlab Biotechnologies) followed by absolute quantitative RT-PCR with the indicated primers (S1 Table).

### Immunoblotting and immunoprecipitation

Cells were lysed in Nonidet P-40 lysis buffer containing 150 mM NaCl, 1 mM EDTA,1% Nonidet P-40, and 1% protease and phosphatase inhibitor cocktail (Roche). Protein concentration was evaluated by Bio-Rad Protein Assay (BioRad). Western blot analysis was performed with the indicated antibodies. For immunoprecipitation, IgG or indicated antibodies (2 μg) were added to cell lysates (1–5 mg) for 4 h at 4 °C and captured by the addition of protein A/G agarose (Pierce) and the precipitates were washed three times with lysis buffer containing 500 mM NaCl as described above. The immune complexes were recovered and subjected to SDS-PAGE and detected by immunoblotting with the appropriate antibodies.

### Site-directed mutagenesis

The template plasmid was amplified with a pair of primers containing a point mutation by TransStart FastPfu Fly DNA Polymerase (Transgen), and the products were subsequently digested with 10 U of DpnI (Takara) at 37 °C for 3 h and were transfected into DH5α competent cells (purchased from Takara Biomedical Technology). Plasmids were extracted with the E.Z.N.A. Plasmid Mini Kit I (Omega).

### Statistical analysis

All data points are expressed as mean values ± standard deviation. Statistical analysis was performed using Origin 9.0 software. All data were analyzed using one-way analysis of variance (ANOVA) with Tukey’s test to determine differences between groups. A value of p < 0.05 was considered statistically significant.

## Supporting information

S1 Fig Screen regulators response to HBV replication. Huh7 cells were transfected with indicated plasmids and pHBV-1.3 for 48 hours. The secretion of HBeAg in the supernatants was measured by ELISA. Bar graphs present means ± SD, n = 3 (**P < 0.01; *P < 0.05), n.s., not significant.
(TIF)

S2 Fig Related to Fig 2. 90K inhibits virus replication. (A) 293T cells were transfected with shRNA-control or indicated shRNA-90K and indicated plasmids for 48 hours prior to western blot analyses. (B and C) A549 cells were transfected with indicated plasmids for 24 hours followed by infection with VSV (MOI = 1) for 24 hours prior to flow cytometry analysis (B) and fluorescent microscopy analysis (C). (D) RD cells were transfected with indicated plasmids for 24 hours followed by infection with EV71 (MOI = 1 or MOI = 10) for 8 hours prior to western blot analyses. (E) Schematic diagram of 90K knockout mice. Murine 90K cDNA includes six exons spanning approximately 9.5 kb. A 7.4-kb fragment of the 90K genomic sequences was replaced with the neomycin resistance gene (neo), leaving 90K exon 1, exon 2, and 30 nt of exon 3 that code for the first 27 amino acids of 90K. (F) WT and *90k*^*-/-*^ MEFs were infected with EV71 (MOI = 1) for 12 hours. The relative protein levels of VP1 (EV71) were quantified by western blot analyses. All experiments were repeated at least three times with consistent results. In the real-time RT-PCR experiments, the control was designated as 1. Bar graphs present means ± SD, n = 3 (**P < 0.01; *P < 0.05), n.s., not significant.
(TIF)

S3 Fig Related to Fig 3. 90K regulates poly (I:C)- and virus-induced inflammatory cytokine expression. (A) A549 cells were transfected with indicated plasmids for 24 hours prior to luciferase assays. (B) HeLa cells transfected with indicated plasmids for 24 hours followed by infection with HSV (MOI = 10) for 6 hours prior to real-time RT-PCR analyses. (C) A549 cells were transfected with indicated plasmids for 24 hours followed by treated with poly (I:C) for 12 hours prior to real-time RT-PCR analyses. (D-F) qRT-PCR analysis of indicated cytokine mRNA in WT and *90k*^*-/-*^ MEFs (D), splenocytes (E), PBMCs (F) treated with poly (I:C) for indicated times. (G and H) The relative levels of IFN and pro-inflammatory cytokines in MEFs (G) and PBMCs (H) infected with 1 MOI of VSV, EV71, ZIKV and 5 MOI of HSV for 12 hours were quantified by real-time RT-PCR. In the real-time RT-PCR experiments, the control was designated as 1. Bar graphs present means ± SD, n = 3 (**P < 0.01; *P < 0.05).
(TIF)

S4 Fig Related to Fig 4. 90K regulated IRF3, IRF7 and NF-κB activation and ISGs expression. (A) A549 cells were transfected with indicated plasmids for 24 hours prior to immunofluorescence assays. The total percentage of p65/50 and IRF3/7 nuclear localization of the whole cells was quantified using ImageJ software and shown as relative percentage of nuclear fluorescence intensity. (B) MEFs of WT and *90k*^*-/-*^ mice were infected with IAV (MOI = 1) for 6 hours prior to immunofluorescence assays. The total percentage of p65/50 and IRF3/7 nuclear localization of the whole cells was quantified using ImageJ software and shown as relative percentage of nuclear fluorescence intensity. (C-E) The relative levels of ISGs in WT and *90k*^*-/-*^ MEFs (C), splenocytes (D) and PBMCs (E) treated with poly (I:C) for indicated times were quantified by real-time RT-PCR. All experiments were repeated at least three times with consistent results. In the real-time RT-PCR experiments, the control was designated as 1. Bar graphs present means ± SD, n = 3 (**P < 0.01; *P < 0.05).
(TIF)

S5 Fig Related to Fig 5. 90K co-localized with TRAF6 and TAK1. (A and B) 293T cells were transfected with the indicated plasmids for 48 hours. Coimmunoprecipitation and immunoblots were performed with the indicated antibodies. (C and D) 293T cells were uninfected or infected with SeV (MOI = 1) for 6 hours prior to immunofluorescence assays. (E) A549 cells were infected with or without SeV (MOI = 1) for 6 hours prior to immunofluorescence assays. (F) A549 cells were transfected with shRNA-ctrl or shRNA-90K plasmid for 24 hours followed by stimulated with poly(I:C) for 12 hours prior to western blot assay. All experiments were repeated at least three times with consistent results.
(TIF)

S6 Fig Related to Fig 6. The BTB and BACK domains of 90K inhibited IAV replication. (A) A549 cells were transfected with indicated truncated 90K constructs for 24 hours followed by infection of IAV (MOI = 1) for 24 hours. The relative levels of NP-specific mRNA was quantified by real-time RT-PCR assay. In the real-time RT-PCR experiments, the control was designated as 1. Bar graphs present means ± SD, (**P < 0.01; *P < 0.05).
(TIF)

S1 Table Primers used for RT-PCR analysis. (DOCX)
