# Supplementary figures and images for: Inducible LGALS3BP/90K activates antiviral innate immune responses by targeting TRAF6 and TRAF3 complex
Source: PLoS Pathog. 2019 Aug 12;15(8):e1008002. doi: 10.1371/journal.ppat.1008002 (PMC6705879; doi:10.1371/journal.ppat.1008002)

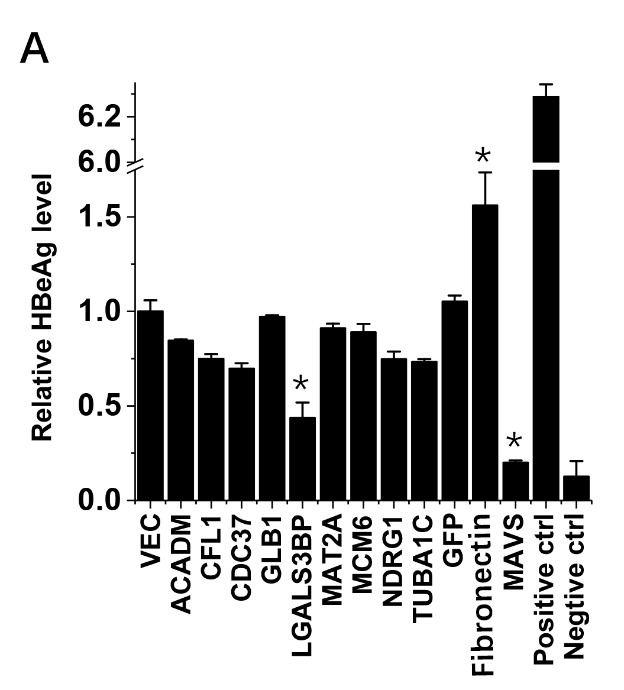

Supplement: S1 Fig — Huh7 cells were transfected with indicated plasmids and pHBV-1.3 for 48 hours. The secretion of HBeAg in the supernatants was measured by ELISA. Bar graphs present means ± SD, n = 3 (**P < 0.01; *P < 0.05), n.s., not significant. (TIF) [file ppat.1008002.s001.tif]

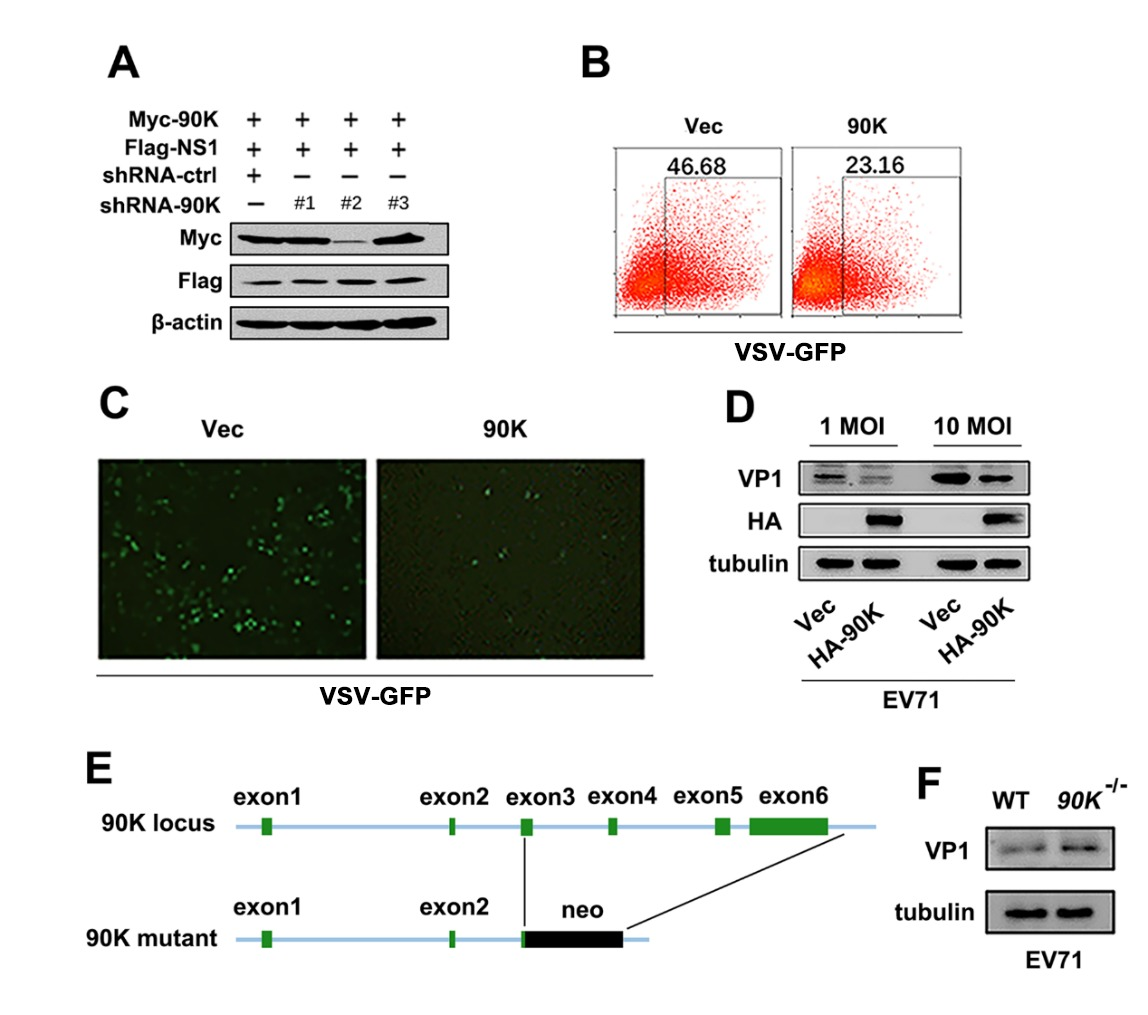

Supplement: S2 Fig — (A) 293T cells were transfected with shRNA-control or indicated shRNA-90K and indicated plasmids for 48 hours prior to western blot analyses. (B and C) A549 cells were transfected with indicated plasmids for 24 hours followed by infection with VSV (MOI = 1) for 24 hours prior to flow cytometry analysis (B) and fluorescent microscopy analysis (C). (D) RD cells were transfected with indicated plasmids for 24 hours followed by infection with EV71 (MOI = 1 or MOI = 10) for 8 hours prior to western blot analyses. (E) Schematic diagram of 90K knockout mice. Murine 90K cDNA includes six exons spanning approximately 9.5 kb. A 7.4-kb fragment of the 90K genomic sequences was replaced with the neomycin resistance gene (neo), leaving 90K exon 1, exon 2, and 30 nt of exon 3 that code for the first 27 amino acids of 90K. (F) WT and 90k-/- MEFs were infected with EV71 (MOI = 1) for 12 hours. The relative protein levels of VP1 (EV71) were quantified by western blot analyses. All experiments were repeated at least three times with consistent results. In the real-time RT-PCR experiments, the control was designated as 1. Bar graphs present means ± SD, n = 3 (**P < 0.01; *P < 0.05), n.s., not significant. (TIF) [file ppat.1008002.s002.tif]

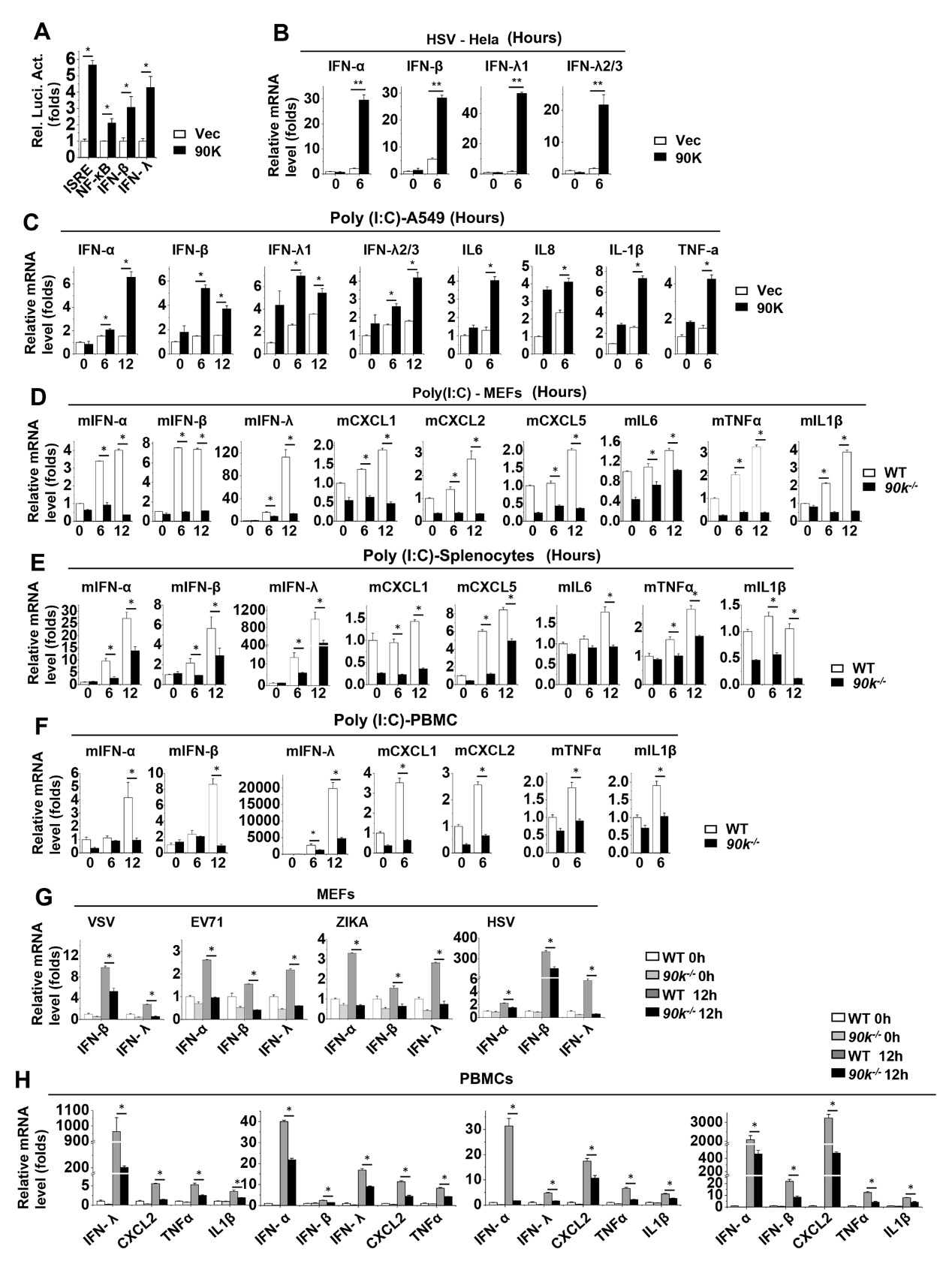

Supplement: S3 Fig — (A) A549 cells were transfected with indicated plasmids for 24 hours prior to luciferase assays. (B) HeLa cells transfected with indicated plasmids for 24 hours followed by infection with HSV (MOI = 10) for 6 hours prior to real-time RT-PCR analyses. (C) A549 cells were transfected with indicated plasmids for 24 hours followed by treated with poly (I:C) for 12 hours prior to real-time RT-PCR analyses. (D-F) qRT-PCR analysis of indicated cytokine mRNA in WT and 90k-/- MEFs (D), splenocytes (E), PBMCs (F) treated with poly (I:C) for indicated times. (G and H) The relative levels of IFN and pro-inflammatory cytokines in MEFs (G) and PBMCs (H) infected with 1 MOI of VSV, EV71, ZIKV and 5 MOI of HSV for 12 hours were quantified by real-time RT-PCR. In the real-time RT-PCR experiments, the control was designated as 1. Bar graphs present means ± SD, n = 3 (**P < 0.01; *P < 0.05). (TIF) [file ppat.1008002.s003.tif]

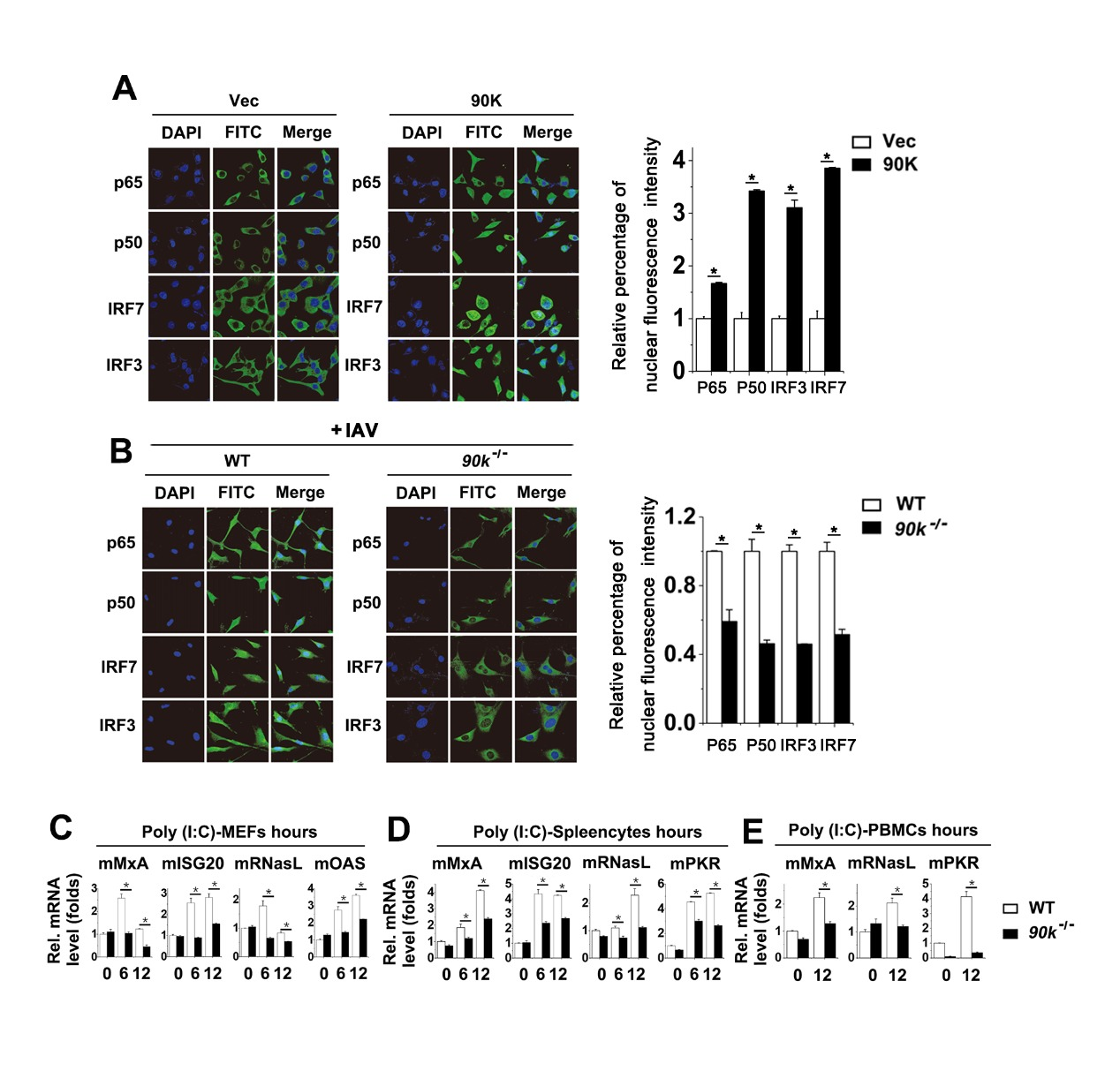

Supplement: S4 Fig — (A) A549 cells were transfected with indicated plasmids for 24 hours prior to immunofluorescence assays. The total percentage of p65/50 and IRF3/7 nuclear localization of the whole cells was quantified using ImageJ software and shown as relative percentage of nuclear fluorescence intensity. (B) MEFs of WT and 90k-/- mice were infected with IAV (MOI = 1) for 6 hours prior to immunofluorescence assays. The total percentage of p65/50 and IRF3/7 nuclear localization of the whole cells was quantified using ImageJ software and shown as relative percentage of nuclear fluorescence intensity. (C-E) The relative levels of ISGs in WT and 90k-/- MEFs (C), splenocytes (D) and PBMCs (E) treated with poly (I:C) for indicated times were quantified by real-time RT-PCR. All experiments were repeated at least three times with consistent results. In the real-time RT-PCR experiments, the control was designated as 1. Bar graphs present means ± SD, n = 3 (**P < 0.01; *P < 0.05). (TIF) [file ppat.1008002.s004.tif]

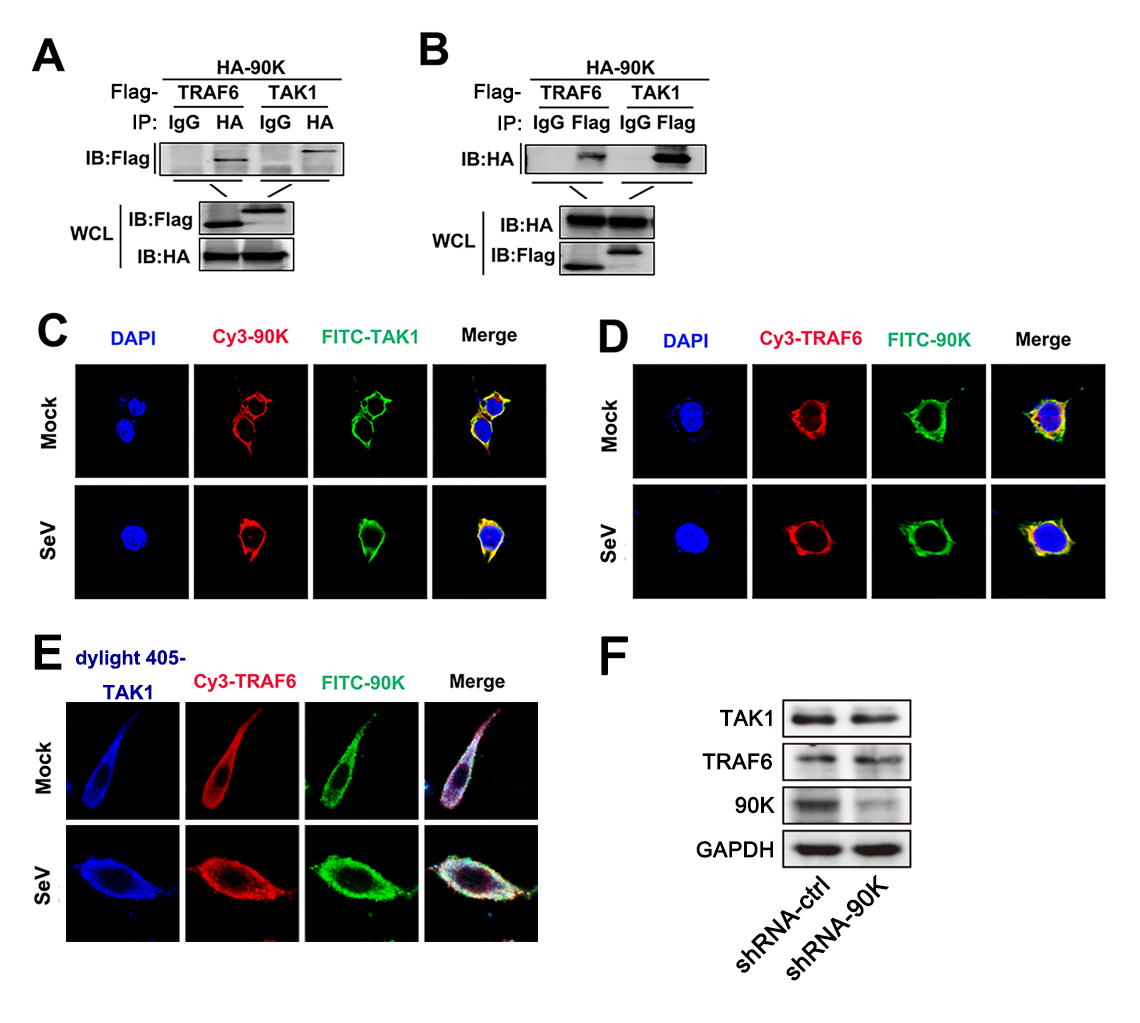

Supplement: S5 Fig — (A and B) 293T cells were transfected with the indicated plasmids for 48 hours. Coimmunoprecipitation and immunoblots were performed with the indicated antibodies. (C and D) 293T cells were uninfected or infected with SeV (MOI = 1) for 6 hours prior to immunofluorescence assays. (E) A549 cells were infected with or without SeV (MOI = 1) for 6 hours prior to immunofluorescence assays. (F) A549 cells were transfected with shRNA-ctrl or shRNA-90K plasmid for 24 hours followed by stimulated with poly(I:C) for 12 hours prior to western blot assay. All experiments were repeated at least three times with consistent results. (TIF) [file ppat.1008002.s005.tif]

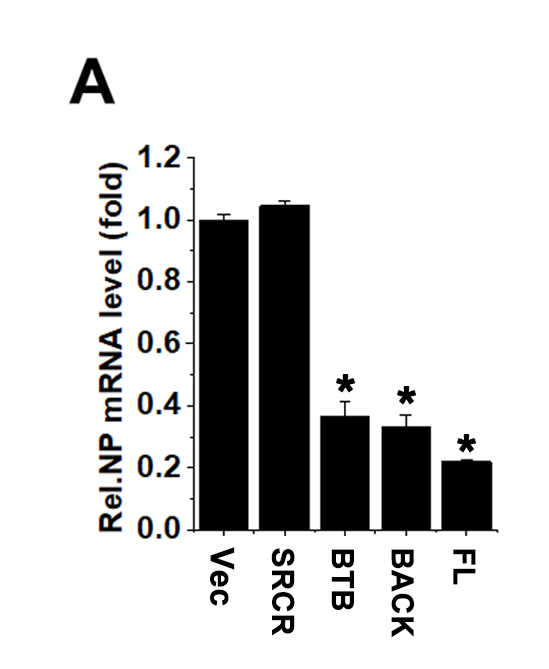

Supplement: S6 Fig — (A) A549 cells were transfected with indicated truncated 90K constructs for 24 hours followed by infection of IAV (MOI = 1) for 24 hours. The relative levels of NP-specific mRNA was quantified by real-time RT-PCR assay. In the real-time RT-PCR experiments, the control was designated as 1. Bar graphs present means ± SD, (**P < 0.01; *P < 0.05). (TIF) [file ppat.1008002.s006.tif]
