# Supplementary material for: Inducible LGALS3BP/90K activates antiviral innate immune responses by targeting TRAF6 and TRAF3 complex
Source: PLoS Pathog. 2019 Aug 12;15(8):e1008002. doi: 10.1371/journal.ppat.1008002 (PMC6705879; doi:10.1371/journal.ppat.1008002)
Supplement: S1 Table — (DOCX) [file ppat.1008002.s007.docx]

**S1 Table. Primers used for RT-PCR analysis.**

| Target | Forward sequence (5‘ to 3') | Reverse sequence (5‘ to 3') |
| --- | --- | --- |
| m*HPRT* | TGCCGAGGATTTGGAAAAAG | CCCCCCTTGAGCACACAG |
| ­m*IFN-α* | ATGGCTAGGCTCTGTGCTTTTCCTC | AGGGCTCTCCAGACTTCTGCTCTG |
| m*IFN-β* | CGTGGGAGATGTCCTCAACT | AGATCTCTGCTCGGACCACC |
| m*IFN-λ2/3* | TCCCAGTGGAAGCAAAGGATTG | TCAAGCACCTCTTCTCGATGG |
| m*IL6* | TGGCTAAGGACCAAGACCATC | TTCTGACCACAGTGAGGAATGTC |
| m*IL1-β* | TCGGACCCATATGAGCTGA | CCACAGGTATTTTGTCGTTGC |
| m*TNF* | GGACTCAAATGGGCTTTCCG | GAGACAGAGGCAACCTGACCAC |
| m*CXCL1* | CAAACCGAAGTCATAGCCACAC | TTGGGGACACCTTTTAGCATC |
| m*CXCL2* | TGAACAAAGGCAAGGCTAACTG | AAGTGAACTCTCAGACAGCGAGG |
| m*CXCL5* | TGGGCAGTGACAAAAAGAAAGC | AAATCCGTGGGTGGAGAGAATC |
| m*Mx1* | TCAAGCCTGGAGTGTCAAGTGCC | AATCTGGAAGCCTTGCCACTGGG |
| m*PKR* | CCTCAGAGAACGTGTTTACG | TCAATTCTGTGTTTCGCTTT |
| m*OAS* | TGTCCTGGGTCATGGTAGTATCAA | TCCCAGATGAGGATGGTGTAGATT |
| m*RNaseL* | GCGAACACATCAATGAGGAAAA | CTGCCTCTGGAACGCTGAG |
| m*ISG20* | CAATGCCCTGAAGGAGGATA | TGTAGCAGGCGCTTACACAG |
| m*LGALS3BP* | TGGAACCTTTTGGATGCCCA | GAAGCCCCGTGGTATCGTT |
| h*GAPDH* | AAGGCTGTGGGCAAGG | TGGAGGAGTGGGTGTCG |
| h*IFN-α* | TTTCTCCTGCCTGAAGGACAG | GCTCATGATTTCTGCTCTGACA |
| h*IFN-β* | AAAGAAGCAGCAATTTTCAGC | CCTTGGCCTTCAGGTAATGCA |
| h*IFN-λ1* | CTTCCAAGCCCACCCCAACT | GGCCTCCAGGACCTTCAGC |
| h*IFN-λ2/3* | TTAAGAGGGCCAAAGATGC | TGGGCTGAGGCTGGATACAG |
| h*IL6* | ACTCACCTCTTCAGAACGAATTG | AGCCATCTTTGGAAGGTTCAG |
| h*IL8* | GGTGCAGTTTTGCCAAGGAG | TTCCTTGGGGTCCAGACAGA |
| h*IL-1β* | CAGAAGTACCTGAGCTCGCC | CATGGCCACAACAACTGACG |
| h*TNF-α* | CTTCTCGAACCCCGAGTGAC | ATGAGGTACAGGCCCTCTGA |
| h*MxA* | GCCGGCTGTGGATATGCTA | TTTATCGAAACATCTGTGAAAGCAA |
| h*PKR* | AAAGCGAACAAGGAGTAAG | GATGATGCCATCCCGTAG |
| h*OAS1* | AGAAGGCAGCTCACGAAACC | CCACCACCCAAGTTTCCTGTA |
| h*LGALS3BP* | GTGAACGATGGTGACATGCG | ATGATGGGGCCTGATCCTTG |
| IAV NP | ATCAGACCGAACGAGAATCCAGC | GGAGGCCCTCTGTTGATTAGTGT |
| HBV capsid-associated DNA | AGAAACAACACATAGCGCCTCAT | TGCCCCATGCTGTAGATCTTG |
| EV71 VP1 | CCCTTTAGTGGTTAGGATTT | CACCAGTTGGTTTAATGGAG |
| VSV nucleoprotein | ATGTCTGTTACAGTCAAGAGAATC | AGAGGAATCTCCTTTGATTTTCTGA |
| HSV DNA | GCTCGAGTGCGAAAAAACGTTC | TGCGGTTGATAAACGCGCAGT |
| ZIKV Envelope protein | CCGCTGCCCAACACAAG | CCACTAACGTTCTTTTGCAGACAT |
